# Water is a preservative of microbes

**DOI:** 10.1111/1751-7915.13980

**Published:** 2021-12-22

**Authors:** John E. Hallsworth

**Affiliations:** ^1^ Institute for Global Food Security School of Biological Sciences Queen’s University Belfast 19 Chlorine Gardens Belfast BT9 5DL UK

## Abstract

Water is the cellular milieu, drives all biochemistry within Earth’s biosphere and facilitates microbe‐mediated decay processes. Instead of reviewing these topics, the current article focuses on the activities of water as a preservative—its capacity to maintain the long‐term integrity and viability of microbial cells—and identifies the mechanisms by which this occurs. Water provides for, and maintains, cellular structures; buffers against thermodynamic extremes, at various scales; can mitigate events that are traumatic to the cell membrane, such as desiccation–rehydration, freeze–thawing and thermal shock; prevents microbial dehydration that can otherwise exacerbate oxidative damage; mitigates against biocidal factors (in some circumstances reducing ultraviolet radiation and diluting solute stressors or toxic substances); and is effective at electrostatic screening so prevents damage to the cell by the intense electrostatic fields of some ions. In addition, the water retained in desiccated cells (historically referred to as ‘bound’ water) plays key roles in biomacromolecular structures and their interactions even for fully hydrated cells. Assuming that the components of the cell membrane are chemically stable or at least repairable, and the environment is fairly constant, water molecules can apparently maintain membrane geometries over very long periods provided these configurations represent thermodynamically stable states. The spores and vegetative cells of many microbes survive longer in the presence of vapour‐phase water (at moderate‐to‐high relative humidities) than under more‐arid conditions. There are several mechanisms by which large bodies of water, when cooled during subzero weather conditions remain in a liquid state thus preventing potentially dangerous (freeze–thaw) transitions for their microbiome. Microbial life can be preserved in pure water, freshwater systems, seawater, brines, ice/permafrost, sugar‐rich aqueous milieux and vapour‐phase water according to laboratory‐based studies carried out over periods of years to decades and some natural environments that have yielded cells that are apparently thousands, or even (for hypersaline fluid inclusions of mineralized NaCl) hundreds of millions, of years old. The term *preservative* has often been restricted to those substances used to extend the shelf life of foods (e.g. sodium benzoate, nitrites and sulphites) or those used to conserve dead organisms, such as ethanol or formaldehyde. For living microorganisms however, the ultimate preservative may actually be water. Implications of this role are discussed with reference to the ecology of halophiles, human pathogens and other microbes; food science; biotechnology; biosignatures for life and other aspects of astrobiology; and the large‐scale release/reactivation of preserved microbes caused by global climate change.

## Graphical abstract

Water acts as a preservative that can to maintain the long‐term integrity and viability of microbial cells, and does this via a variety of mechanisms. Assuming that the components of the cell membrane are chemically stable, or at least repairable, and the environment is fairly constant, water molecules can apparently maintain membrane geometries over very long periods provided these configurations represent thermodynamically stable states. Currently, a large‐scale release/reactivation of preserved microbes is being caused by global climate change.

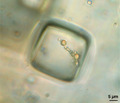

## Introduction

Water is one of the most versatile and protean of substances. To the biologist, it seems that there are few roles that this substance cannot and does not perform: water is implicated in the origin of life (Miller and Urey, 1959) and is the very fabric of life. Furthermore, across much of the Earth, water seems omnipresent. It forms the oceans, lakes and rivers; pervades in the atmosphere as vapour, clouds and fog; occurs as permafrost, glaciers, sea ice and the polar ice sheets; and forms subsurface aquifers, groundwaters and water flows. Water also provides for the hydration of soils and sediments, formation of thin films or dew on surfaces, evaporative processes, precipitation and other aspects of the hydrological cycle. All of the biosphere’s biochemistry occurs within these aqueous milieux, including decay processes and nutrient cycling. However, one aspect that receives scant attention is whether water is necessarily a liability for inactive microbes or whether in some cases it helps to maintain cellular integrity.

Life as we know it on Earth evolved in, and is sustained by, water (Ball, 2017). What is more, this truism applies at all scales. Cellular systems, and their macromolecular components, derive their structural–functional properties from water, and Earth is habitable due to the abundance and liquidity of its water. For Earth’s life forms, water is the cellular solvent; a reactant; provides hydration for biomacromolecules; enables ionization of salts; facilitates macromolecular stability, flexibility and cohesion; provides cell turgor; and more (Daniel *et al*., 2004; Ball, 2008). In addition, entropically unfavourable interactions between hydrophobic chemical groups and water molecules drive the self‐organizing behaviour seen in biological systems.

Water facilitates transport of substances into and out of the cell, including nutrients and waste products. Microbial cells also compete with each other and communicate with cells of their own kind and other microbes/organisms via soluble and hydrophobic substances released into the aqueous extracellular milieu (Cray *et al*., 2013a; Combarnous and Nguyen, 2020). At a biosphere level, water is in constant exchange between the Earth’s biomass and its hydrological cycle, and fluxes of water move between organisms (Nelson *et al*., 2020; Pérez‐Ruiz *et al*., 2021). Trophic interactions are also facilitated by water at many levels and via a diversity of mechanisms, including the infection processes of microbial pathogens (e.g. Hallsworth and Magan, 1994; Foster *et al*., 2017).

Cellular systems can satiate part of their water requirements by generating H_2_O via their own metabolic processes (Kreuzer‐Martin *et al*., 2005, 2006; Li *et al*., 2016). However, it is the thermodynamic parameter *water activity* (the effective concentration of water molecules) which determines whether biochemical and biological processes can occur. The water‐activity scale extends from a minimum of 0 (equivalent to 0% relative humidity) to 1, for pure water (equivalent to 100% relative humidity). This parameter is dependent on temperature and pressure, as defined by Raoult’s law. Water activity limits the biological functions of individual microbes and ultimately limits the boundaries of life on Earth (Brown, 1976; Grant, 2004; Stevenson *et al*., 2015a, 2017).

Any factors that perturb the interactions between water and the cell can impose energy‐expensive inhibitory stresses on microbial systems. These stresses can be damaging to cellular structures and frequently are lethal. Water‐mediated stresses can be caused by factors such as dilution or hypo‐osmotic shock; salts and other solutes, or hyper‐osmotic shock; supra‐ or sub‐optimal water activity; organic solvents; temperature changes or extremes; dehydration–rehydration events; and freeze–thawing cycles. Chaotropic substances (those which entropically disorder biomacromolecules) also induce a water‐mediated cellular stress and render some environments unhabitable and thereby limit the extent of Earth’s biosphere (Hallsworth *et al*., 2007; Cray *et al*., 2015).

Given that water and water‐mediated stresses can be lethal to microbial cells, it seems counterintuitive that water may also function as a potent preservative of cell integrity and viability for microbial communities. This article considers the evidence that water is indeed a preservative, conserving living microbial cells over long timescales where conditions do not permit cell metabolism.

## Pure liquid water, freshwater and seawater as preservatives

### Distilled water

Independent research groups have reported on the longevity of microbial cells that have been stored in distilled water over periods of years or decades. For example, Iacobellis and DeVay (1986) inoculated sterile distilled water with cells of plant‐pathogenic isolates of *Pseudomonas syringae* subsp. *syringae* and then stored them at 10°C. Whereas there was a decrease in total viable cells during the initial months, the remaining cells underwent adaptative changes, including ultrastructural modifications, and a high percentage of these survived in a viable condition throughout the 24‐year study period. Furthermore, pathogenicity towards the plant host was retained. Studies into bacterial viability stored cells in distilled water for 13–20 years revealed that their viability is also maintained (Iacobellis and DeVay, 1986; Liao and Shollenberger, 2003).

Experiments in which phylogenetically diverse fungi stored in either distilled water or aqueous media (with a water activity of ~ 1) have yielded similar results (Burdsall and Dorworth, 1994; Elliott, 2005; Castro‐Rios and Bermeo‐Escobar, 2021). For example, Elliott (2005) stored biomass of five strains of the plant‐pathogenic fungus *Gaeumannomyces graminis* var. *graminis* for a period of 10 years in sterile deionized water, mineral oil and on dried paper at ~ 24°C. Biomass was also stored in a 40% (v/v) glycerol solution at −20 and −75°C. Whereas all strains survived throughout the 10‐year period in the water and mineral oil treatments, those on dried paper survived for only ≤ 6 months; those in glycerol at −75°C survived for 0 and 48 months; and those in glycerol solution at −20°C did not survive at all (see Table III of Elliott, 2005). Water was a superior preservation method when compared with either paper‐ or low‐temperature glycerol storage. Furthermore, cells within mineral oil were likely contained within an aqueous extracellular layer so they too were effectively preserved in water. Interestingly, at 4°C survival was poor in water (or mineral oil), a phenomenon which may relate to changes in the density of water—and associated cellular damage—that are peculiar to this temperature (Pavankumar *et al*., 2021). In general, however, water has proved the most effective preservative of fungi in studies where comparisons were carried out (for further examples, see the review of Castro‐Rios and Bermeo‐Escobar, 2021).

For the overwhelming majority of microbes, the stability and functionality of their biomacromolecular systems, their cellular metabolism and their ability to proliferate are all optimal at high water activity, in the range of 0.950 to 1 (see figure 2 of Stevenson *et al*., 2015b; for lower water‐activity limits for growth, see table 1 of Brown, 1976). The cells of some extremely xerophilic microbes (microorganisms that grow optimally in low water‐activity conditions, including halophiles) are unstable in pure water or seawater, the latter of which has a relatively high water activity of 0.980 (Benison *et al*., 2021). However, less than 1% of microbes are in this category.

Oxidative damage can increase in desiccated cells (see below), and active metabolic/growth processes generate reactive oxygen species which cause oxidative damage (Hallsworth, 2018). Therefore, the hydrating conditions for, and the low‐or‐zero growth rate of, cells in pure water mitigate against these problems. Cells in a state of zero growth include those starved of nutrient sources; those in biophysically extreme conditions (e.g. at temperatures or water activities beyond their windows for cellular metabolism); and those entering or leaving a dormant desiccated state known as anhydrobiosis. Nevertheless, such cells may retain enzymatic basal functions such as the ability to repair DNA damaged by certain toxic and stressful chemicals or reactive oxygen species (Bosch *et al*., 2021; Shoemaker *et al*., 2021). For lipids within the cell membrane, the avoidance of entropically unfavourable interactions with water molecules maintains hydrophobic structural interactions and motifs (Ball, 2008) thereby stabilizing cellular structures (see *Cytosol‐lipid bilayer‐extracellular solution; an aqueous continuum* section of McCammick *et al*., 2010). Long‐term immersion in water also mitigates against biocidal events/factors because it avoids potentially lethal desiccation–rehydration events (see below), and at depth in aquatic microbial habitats can filter out damaging solar ultraviolet radiation, as explained below.

Whereas it is not claimed here that *all* microbes are preserved effectively in water, it is nevertheless remarkable that pure water can be highly effective for long‐term preservation of some microbial cells, especially for those species that are fast‐growing, cultivatable copiotrophs and in some cases are pathogens that exhibit interactions with higher organisms. These microbes (see Iacobellis and DeVay, 1986; Liao and Shollenberger, 2003) were not isolated from low‐nutrient (oligotrophic), low‐temperature freshwater habitats that select for ability to survive in water while inactive. In the natural environment, freshwater habitats that are perpetually cold and/or nutrient‐depleted do occur throughout much of the biosphere, for example, many Antarctic lakes. Microbial life cannot readily proliferate (or do so only over long time periods) in some of these environments and rates of metabolism can be so slow as to be undetectable, yet cells appear to survive there over extended, indeterminate timescales (see below).

### Microbial longevity in freshwater and seawater

There are diverse lines of evidence indicating that the cells of some microbes survive in bodies of freshwater and seawater for very long periods of time. In deeply buried marine sediments, the low‐energy conditions often dictate that microbial cells subsist under strong energy‐ and nutrient limitation for hundreds to thousands of years, and possibly even longer (Hoehler and Jørgensen, 2013; Jørgensen and Marshall, 2016). Temperatures and pressures tend to remain constant, but there is some background ionizing radiation in these sediments. However, theoretical calculations (based on microbes located in ice) indicate that some cells—capable of self‐repair even when they appear to be otherwise inactive—can survive this radiation for millions of years (Price, 2009). Therefore, cellular macromolecules such as nucleic acids and lipids, and an intact cell membrane, can serve as indicators for viability in the deep biosphere where, under some environmental conditions, they would disintegrate if a living cell was not maintaining them. Many cells in subsurface sediments that are thousands of years old may be in a state of near‐zero metabolism (known as cryptobiosis or anabiosis) and have intact ribosomal RNA that can be hybridized with fluorescent oligonucleotide probes (Buongiorno *et al*., 2017). Individual cells within such habitats are likely to be ancient, although acetate from pyrogen and H_2_ may support some low‐level metabolism and very occasional cell division in such populations.

The Antarctic freshwater Lake Vostok is several thousands‐years‐old, ice‐sealed (subglacial) and dark, at subzero temperatures and high pressure, and nutrient‐depleted, yet the water of the main basin contains a sparse community of microorganisms (Siegert *et al*., 2001; Bulat, 2016; Gura and Rogers, 2020). Under these conditions, it is likely that their cells survive in an inactive (or virtually inactive) state for vast time periods, as do those found in some seawater‐ and brine environments (see below). The microbes in Lake Vostok include fungi, *Actinobacteria*, *Alphaproteobacteria*, *Betaproteobacteria*, *Gammaproteobacteria* and *Firmicutes* (Gura and Rogers, 2020). Other Antarctic freshwater lakes host primitive life, including the ice‐covered, ultra‐oligotrophic 0.5 to 5°C Lake Untersee (Marsh *et al*., 2020). This lake, thought to have been sealed by its ice cover for 100,000 years, contains viable prokaryotic communities including ancient archaean stromatolites (Andersen *et al*., 2011; Mulyukin *et al*., 2014). Many such freshwater and marine environments are stable over geological time periods (with very low, if any, perturbation) and the turnover of microbial biomass ranges from years to thousands of years according to calculations of the racemization between L‐ and D‐amino acids within necromass (Lomstein *et al*., 2012; Mhatre *et al*., 2019).

A recent study of the environmental persistence of heterotrophic taxa of bacteria, originally soil isolates but tested for survival in water, was carried out by sealing populations of each in an aqueous liquid (phosphate‐buffered saline) and storage in the dark at 25°C for 1000 days (Shoemaker *et al*., 2021). All of the 100 populations of cells (21 bacterial taxa) survived for 1000 days when stored under extreme energy limitation in this way. Based on growth data (there was some cell death, but the necromass was used by remaining cells), the authors calculated the mean time that would be taken for extinction of an individual cell and for each population (figure 2 of Shoemaker *et al*., 2021).

Based on this analysis, the populations of some of the water‐stored cells and populations will likely remain viable for mean times of up to 10^2^ days and 10^5^ years, respectively, so, in some cases considerably longer. Given the turnover of necromass in this study, cell longevity was driven in part by production of new cells rather than an inability of remaining cells to survive. In some natural environments, such as oligotrophic Antarctic lakes, energy and nutrient limitation combined with what Shoemaker *et al*. (2021) call an initial ‘purifying selection’ (selection for those cells capable of survival) will lead to indefinite survival times for both individual cells and their populations.

Microbial cells persist with growth rates near zero according to estimates of total energy flux in deeply buried sediments yet the community may be turning over, albeit at a near‐imperceptible rate (Hoehler and Jørgensen, 2013; LaRowe and Amend, 2015). Cells in deep sediments are apparently alive because they are intact (e.g. in sub‐seafloor sediment; Cragg *et al*., 1996), have intact mRNA (as is also shown in studies of permafrost; see below) that is consistent with available metabolites (e.g. in sub‐seafloor sediment; Bird *et al*., 2019) and in some cases have even been cultured (e.g. from sub‐seafloor sediment; Imachi *et al*., 2014; Morono *et al*., 2020). Heterotrophic bacteria persist in 33.5–104 million‐year‐old sub‐seafloor basalt (Suzuki *et al*., 2020), and sensitive isotope‐labelling experiments have been done on deep sub‐seafloor sediments using radio‐labelled carbon‐ or nitrogen substrates or deuterated water along with subsequent detection of substrate assimilation by cells (Morono *et al*., 2011; Trembath‐Reichert *et al*., 2017). The results showed that up to 76% of all the cells present had an active (albeit slow) anabolic metabolism, so are by definition, alive.

## Microbial life within water ice

Freeze–thawing can kill microbial cells depending on factors such as the rate of cooling and type of microbe or physiological condition (Park *et al*., 1997; Meisner *et al*., 2021). However, low‐temperature environments such as snow and water ice are habitats for microbes; ice environments can also preserve microbes and other organisms in an inactive‐yet‐viable state (induced primarily by low temperature and termed cryobiosis) (Price, 2000; Maccario *et al*., 2015; Hallsworth, 2021). The low‐temperature (low‐entropy) conditions contribute to the stabilization of the cellular structure/ultrastructure, yet this preservation process (whether in frozen or non‐frozen cells) is mediated by water given that the cell membrane and other biomacromolecules are hydrated. In addition, the survival of freezing and thawing events are dependent on the properties and activities of the cell’s water (Calcott and MacLeod, 1975a,b; Kruuv *et al*., 1978; Tanghe *et al*., 2004).

Viable cells have been recovered from ice cores around the world (Christner *et al*., 1999, 2003), including those of 120 000‐year‐old Greenland ice (Miteva *et al*., 2004), according to culture‐based studies using low‐nutrient growth media. Direct detection of viable endospores in ice‐core samples has also been achieved from measurements indicating the release of dipicolinic acid (a substance unique to endospores) (Yung *et al*., 2007). Even flowering plants have been revived from from fruits preserved in permafrost for tens of thousands of years (Yashina *et al*., 2012).

Microbial cells have been also recovered from ice known to be 750 000 years old (Christner *et al*., 2003) and from possibly the oldest ice on Earth (Bidle *et al*., 2007) dated at approximately 8 million years (Sugden *et al*., 1995), although the exact age has been debated (Ng *et al*., 2005). Studies have shown that permafrost frozen for millions of years harbours intact microbial cells containing DNA and mRNA that also show other evidence of metabolism and can be cultivated (Rivkina *et al*., 1998; Orsi *et al*., 2013; Coolen and Orsi, 2015; Liang *et al*., 2019, 2021; Pedrós‐Alió, 2021; Sipes *et al*., 2021). Such cells are apparently capable of critical survival‐related processes, such as DNA repair during reactivation process and possibly even during their long‐term preservation in an inactive or near‐inactive state (Price, 2009; Liang *et al*., 2021).

Whereas permafrost is a convenient model to study the long‐term survival of microbes in natural environments, the cryosphere is a large‐scale and diverse repository of microorganisms that includes those in the polar ice caps, glacial ice, and sea ice. For examples of ancient microbes recovered from such environments (and a discussion of ice‐sterilization protocols), see Pedrós‐Alió (2021). When microbes freeze, the formation of ice within the cell can threaten cellular integrity because ice crystals can puncture and rupture membranes mechanically (Muldrew and McGann, 1990, 1994). Alternatively, growth of ice crystals within the cell membrane interferes with lipid–lipid interactions. If cooling occurs quickly, however, such ice crystals do not form, the cell membrane remains intact, and cells can survive (Calcott and MacLeod, 1975a).

Low‐temperature, low‐entropy conditions also act to stabilize/preserve cellular membranes and other cellular macromolecules. Whereas the limit for metabolic processes in cells of extreme psychrophiles is about −40°C (Price and Sowers, 2004; Panikova *et al*., 2006; de Vera *et al*., 2014), many microbes survive storage at −196°C in liquid nitrogen (Tsutsaeva *et al*., 2008) and there is no known lower limit to the temperatures at which cells can remain intact and viable. In addition to the physical damage that ice crystals can cause, solutes become more concentrated under freezing conditions, leading to osmotic stress and dehydration of the cell (Clarke *et al*., 2013; Clarke, 2014). Extreme dehydration drives vitrification of the cytosol, which prevents the diffusion of gases and metabolites (Clarke, 2014; Bakermans, 2017). This complete suspension of life processes by solid H_2_O at low subzero temperatures helps to maintain the viability of cells, many of which are also capable of surviving the thawing process.

## Aqueous brines conserve cell viability

Synthetic brines can be made using salts plus compounds such as ethylene glycol, propylene glycol (Sahoo *et al*., 2017), glycerol (Takamura *et al*., 2012), methanol (Bui *et al*., 1997) and hexane + ethanol (Cuiec *et al*., 1994). Within Earth’s biosphere, however, the brines that host microbial communities are typically formed from the dissolution of ionic solutes in water. The chemistry, biophysics and habitability of these aqueous brines can vary greatly. Some are highly permissive for active (halophile) life when temperatures and nutrient availability permit, most notably NaCl brines such as those of crystallizer ponds (Grant, 2004; Daffonchio *et al*., 2006; Lee *et al*., 2018), but others are not; particularly, chaotropic ones such as the deep‐sea MgCl_2_‐saturated Lake Discovery (Hallsworth *et al*., 2007; Belilla *et al*., 2019; Hallsworth, 2019; Benison *et al*., 2021; Sanz *et al*., 2021).

Nevertheless, chemically diverse brines can preserve microbial cells in a viable condition and/or conserve their biomacromolecules including DNA and cell membrane (see the studies of Duda *et al*., discussed below). Both ions and biomacromolecules have hydration shells and so this preservative effect is mediated by water, rather than solely by the salt (Ball and Hallsworth, 2015; Hibino *et al*., 2017; Pachler *et al*., 2019; Smith and Smith, 2020). Ions within the microbial cell always remain hydrated. If they were not, and whereas it may be difficult to imagine the alternative (i.e. ‘naked’ water‐less ions) because they do not exist in an aqueous solution, the intense electrostatic fields of ions such as Mg^2+^ and Ca^2+^ would be highly damaging to the cell’s macromolecules. This is because one of the arguably biophilic aspects of water is that it is highly effective at electrostatic screening, due to its relatively high dielectric constant. Without this property, anything ionic would likely to attach to anything counterionic (Maurer and Oostenbrink, 2019). In reality, the cell is shielded from such damage by water molecules that participate in the hydration of both ions and biomacromolecules.

### NaCl (including thalassohaline) brines

In natural environments, NaCl brines typically host high‐biomass and phylogenetically diverse microbial ecosystems that are highly active, whether located at the surface or within the subsurface (Lee *et al*., 2018). This is because their water activity is permissive for the metabolism, proliferation and ecological interactions of numerous halophilic organisms (Stevenson *et al*., 2015a; Lee *et al*., 2018). However, some such brines can also limit or even prevent metabolic activity and cell division; most notably under oligotrophic conditions and/or at low temperature. And yet, even under these conditions, live cells are preserved.

**Fig. 1:**
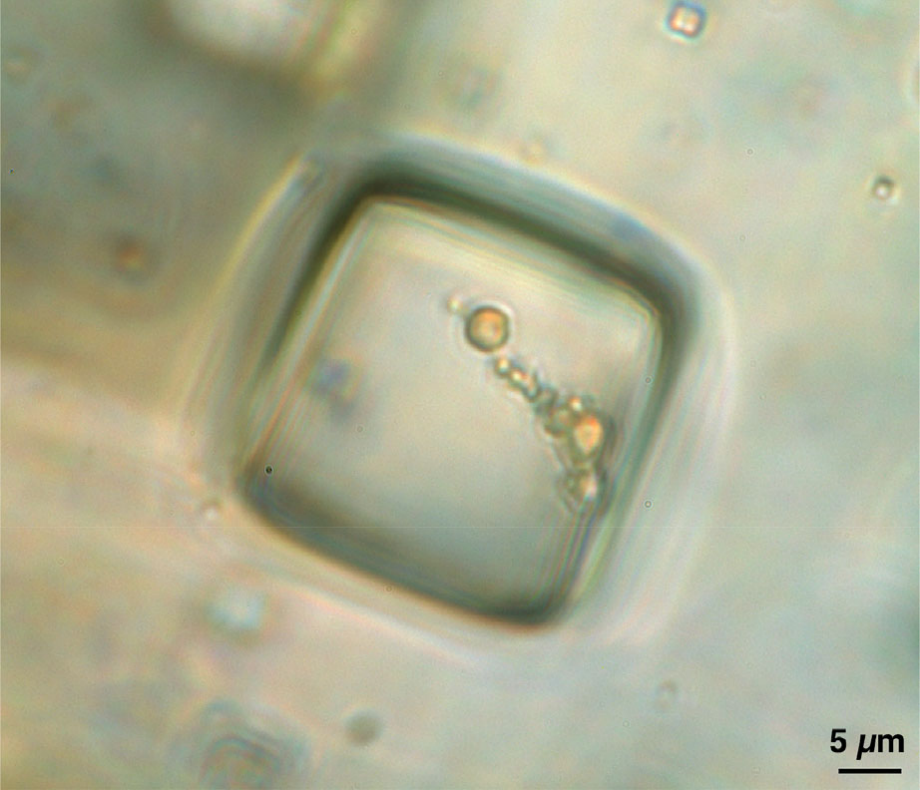
Primary fluid inclusion in bedded halite from the Permian Nippewalla Group, at a depth of 429 m the subsurface of Kansas, USA (~ 267 million years old, based on paleomagnetic dating of the rocks in a nearby core: Foster *et al*., 2014). The micrograph was taken under plane‐transmitted light, by Kathleen C. Benison (West Virginia University, WV, USA). The spheres, which fluoresced blue and green under UV‐visible light (not shown here), appear to be phylogenetically diverse microbial cells.

Some relatively short‐term (laboratory‐based studies) have been carried out that confirm that microbes survive preservation for years or decades in experimentally produced hypersaline inclusions (Dombrowski, 1966; Elabed *et al*., 2019); even cells of non‐halophiles can survive in hypersaline inclusions of mineralized NaCl (halite) and remain viable according to one study carried out over a 14‐year period (Elabed *et al*., 2019). In nature, this phenomenon is best illustrated by the microbes found preserved within the hypersaline fluid inclusions of halite (Fig. 1; Fendrihan *et al*., 2012; Lee *et al*., 2018; Benison, 2019; Afouda *et al*., 2020) or hypersaline—subzero‐yet‐liquid—cryopegs within permafrost (Colangelo‐Lillis *et al*., 2016; Afouda *et al*., 2020; Rapp *et al*., 2021).

#### Could cells be preserved for hundreds of millions of years?

Microbes have been reported, and in many cases cultured, from brine inclusions of halite that are typically extremely energy‐/nutrient‐limited. These include brine inclusions of Pleistocene mineralized NaCl (halite) (9,600 to 97,000 years old) (Mormile *et al*., 2003; Schubert *et al*., 2010) and Permian halite (250 to 270 million years old; Fig. 1; Vreeland *et al*., 2000) as discussed by Schreder‐Gomes *et al*. (2021, 2021). Microbial cells have been recently observed in Precambrian fluid inclusions of halite that is 830 million‐years‐old based on radiometric dating of overlying and underlying igneous rocks (Schreder‐Gomes and Benison, 2021, 2021; Schreder‐Gomes *et al*., 2021, 2021), albeit that attempts have not yet been made to culture them. For a discussion of the robustness of dating of these inclusions, which were identified as primary inclusions which formed when the halite originally precipitated, see Supporting Information Appendix S1. At this time, 830 million years ago, early plant life was developing on Earth but it is thought that the first protozoan had not yet appeared (Cunningham *et al*., 2017). It is not possible to ascertain whether the cells in these Precambrian fluid inclusions underwent any cell division(s) during their entrapment. However, there was no visible evidence of this (Schreder‐Gomes *et al*., 2021, 2021), halophiles have a cell membrane composition that is most thermodynamically stable at high salt concentrations (Kates, 1993; Gunde‐Cimerman *et al*., 2018), and the constrained conditions dictate that these cells must be ancient.

#### Low‐temperature NaCl brines

Cold NaCl brines also host microbial life. Whereas the low‐temperature limit for psychrophile metabolism appears to be near –40°C (see above), most halophiles are unable to grow or retain metabolic activities at low temperatures. The lowest temperatures at which microbial cell division has been observed are in the range of –10 to –18°C (for references, see table 2 of Rummel *et al*., 2014 and figure 1 of Lee *et al*., 2018). However, no active life has ever been recorded at such low temperatures in NaCl‐saturated brines. In general, most psychrotolerant halophiles are inactive at ≤ 0°C whether under anaerobic or aerobic conditions (see figure 2b of Harrison *et al*., 2015), but cells of these same organisms are not necessarily killed if temperatures decrease to values below the growth window (indeed, halophiles can be stored in liquid nitrogen).

The lowest temperature ever recorded on Earth is −89.2°C and many NaCl brines occur in locations or at times with temperatures below −40°C. Sea‐ice brine channels of the polar regions and the hypersaline Deep Lake (Vestfold Hills, Antarctica) are subject to environmental temperatures that oscillate diurnally, seasonally and, according to prevailing climatic conditions, can reach −20°C or even below. High salt concentrations, however, prevent brines from freezing so cells remain suspended in the aqueous liquid. Cells in such habitats are active if temperatures and nutrients permit, but remain viable yet inactive when biophysical conditions or nutrients are unavailable (Mou *et al*., 2012; Williams *et al*., 2017; Cooper *et al*., 2019; Karan *et al*., 2020; Rapp *et al*., 2021). Genome‐based functional analyses of microbes within the cryopeg brine of a 40 000‐year‐old permafrost identified *Marinobacter* as a dominant taxon within the community (Rapp *et al*., 2021). Both studies of laboratory‐synthesized brine inclusions and those that focus on *in situ* ecology have revealed the complexity and dynamics of halophile communities that subsist or survive in these habitats (Colangelo‐Lillis *et al*., 2016; Huby *et al*., 2021; Najjari *et al*., 2021; Rapp *et al*., 2021). Other studies have focused on organic compounds within inclusions of halite (Chan *et al*., 2018) (and billions‐of‐years‐old fluid inclusions of quartz—mineralized silicon dioxide— Schreiber *et al*., 2017, and baryte—mineralized barium sulfate—Mißbach *et al*., 2021). In summary, NaCl brines are known to preserve simple organic compounds, complex macromolecules and entire cells over extraordinary time periods.

### Acid brines

Acid brines, such as those found in surficial lakes and groundwaters of Western Australia, are biologically hostile environments yet they contain communities of halophilic or polyextremophilic microbes according to observations of foam production (Benison, 2008), metagenomic surveys (Mormile *et al*., 2009; Zaikova *et al*., 2018) and lipid analyses (Johnson *et al*., 2020). These brine lakes cycle between flooding (at which time their waters are diluted) and evapo‐concentration, when their salt concentration, water activity and pH become more extreme. A recent study of the water activity of several acid lakes of Western Australia revealed water‐activity values for evapo‐concentrated brines as low as 0.714 (for the pH‐1.4 brine of Gneiss Lake; Benison *et al*., 2021).

Whereas metagenomic analyses of brine from the evapo‐concentrated Gneiss Lake revealed diverse microbial taxa, there was no direct evidence of metabolic activity or microbial growth in these waters (Johnson *et al*., 2015), nor has there been for microbes under these conditions in either other natural environments or laboratory culture media (Harrison *et al*., 2015; Stevenson *et al*., 2015a; Benison *et al*., 2021; Hallsworth *et al*., 2021a). Collectively, these studies show that acid brines preserve DNA and facilitate microbial activity when flooded/diluted and suggest that cells may survive even during the evaporation stages of such lakes. Microbes are also preserved within the acid‐brine inclusions of halite formed during the evaporation of acid brines, at some time point(s) at least (Benison, 2019). However, more work is needed to establish whether viable cells are preserved at all stages of evapo‐concentration.

### Chaotropic brines

Like acid brines, chaotropic brines are hostile to life, including those rich in MgCl_2_ (Hallsworth *et al*., 2007; Yakimov *et al*., 2015), CaCl_2_ (Cray *et al*., 2013b; Tregoning *et al*., 2015) and LiCl (Cray *et al*., 2013b; Rubin *et al*., 2017). Other salts—used in molecular protocols or microbiology experiments—are also known to be chaotropic, including ammonium nitrate, guanidine hydrochloride, guanidine thiocyanate, iron chlorides, perchlorates, potassium iodide and sodium thiocyanate (Cray *et al*., 2013b; Alves *et al*., 2015).

So‐called ‘bittern’ brines that derive from evaporated seawater and are enriched in MgCl_2_ (because the NaCl precipitates first), and Dead Sea brine—which is also high in MgCl_2_—do not permit microbial growth or metabolism once the concentration of this chaotrope goes beyond a critical level that cannot be tolerated by the cellular system (Javor, 1984; Oren, 1998; Hallsworth *et al*., 2007; Bodaker *et al*., 2010; Khlaifat *et al*., 2010; Yakimov *et al*., 2015). The salt MgCl_2_ decreases water activity, increases cell turgor, has a low pH and chaotropically damages cellular structures (Hallsworth *et al*., 2007; Alves *et al*., 2015). Many halophile species can grow in the water‐activity range 0.801 to 0.845 in NaCl‐dominated brines (Stevenson *et al*., 2015a, 2017; Lee *et al*., 2018), which is the range for which mRNA data indicate that all microbial activity ceases in the MgCl_2_‐dominated brines of Lake Discovery and the nearby deep‐sea Lakes Kryos and Hephaestus—about 2.4 to 3 M MgCl_2_ (Hallsworth *et al*., 2007; Yakimov *et al*., 2015; La Cono *et al*., 2019). Diverse lines of evidence, including data showing that addition of salts that entropically stabilize biomacromolecular structures (kosmotropic salts) to MgCl_2_‐rich brines, up to about 3 M MgCl_2_, enables some microorganisms to resume metabolic activity, and functional assays of cellular macromolecules, confirm that chaotropicity is the limiting stress parameter (Hallsworth *et al*., 2007; Alves *et al*., 2015; Yakimov *et al*., 2015; La Cono *et al*., 2019).

Whereas culture‐based studies, mRNA‐based studies and macromolecular assays all indicate that cellular activity does not/cannot occur beyond 3 M MgCl_2_, in most cases the MgCl_2_ concentration limits for metabolic activity or cell division are considerably lower, whether in deep‐sea MgCl_2_ brines or MgCl_2_‐rich culture media (Hallsworth *et al*., 2007; Alves *et al*., 2015; Stevenson *et al*., 2015a; Yakimov *et al*., 2015; La Cono *et al*., 2019)^1^. Studies on the activities of other chaotropic salts (guanidine hydrochloride and guanidine thiocyanate) against cells of phylogenetically diverse bacteria and yeast indicate that these salts not only inactivate microbes, but that they damage and kill cells yet preserve their structures with the cell membrane intact (Duda *et* *al*., 2004, 2005). The CaCl_2_‐saturated Don Juan Pond (Antarctica) brine is also uninhabitable (for a discussion, see Stevenson *et al*., 2015b), and culture‐based studies carried out using lower CaCl_2_ concentrations indicate that the mode‐of‐inhibitory action is similar to that of MgCl_2_ (Alves *et al*., 2015).

Whereas it is clear that chaotropic brines can be lethal, culture‐based studies record growth/cell division only up to concentrations of about 2 M MgCl_2_ (Hallsworth *et al*., 2007; Alves *et al*., 2015; Stevenson *et al*., 2015a; Jančič *et al*., 2016). For some halophiles, metabolism only ceases at about 2.4 to 3 M MgCl_2_ (see above), so we know that MgCl_2_ brines can preserve cellular structures, at least up to those concentrations, for a small number of taxa and under particular environmental conditions (Yakimov *et al*., 2015). It is also established that a 5‐M MgCl_2_ brine selectively preserves some nucleic acids (Hallsworth *et al*., 2007). A study of surface‐brine Dead Sea samples that had been stored for 56 years (since 1936) revealed that cells were preserved in a viable and culturable state, based on the isolation and phenotypic characterization of 158 isolates of *Archaea* (Arahal *et al*., 2000). Whereas salts were not analysed in this study, the MgCl_2_ concentration of Dead Sea surface brine at that time was 142 g l^‐1^, which is ~ 1.5 M (Elazari‐Volcani, 1940).

Moderate cellular stress, including that induced by chaotropic substances, can stimulate cellular vitality through various responses and adaptations, such as increasing the rate of energy generation (Hallsworth *et al*., 2003; Fallico *et al*., 2011; Alves *et al*., 2015; Hallsworth, 2018). Furthermore, responses and adaptations to specific stress parameters confer resistance to other sources of stress. For example, the same protein‐stabilization proteins upregulated by chaotropicity (Hallsworth *et al*., 2003) also increase tolerance to temperature extremes and other stresses; the accumulation of compatible solutes that function in osmotic adjustment in response to salts (Grant, 2004; Alves *et al*., 2015; Gunde‐Cimerman *et al*., 2018) can also protect macromolecules and other cellular structures against other (mechanistically diverse) stresses; and oxidative stress responses induced by chaotropicity (Hallsworth *et al*., 2003) mitigate against reactive oxygen species generated via other mechanisms. Extreme chaotropicity also eliminates potential grazers and predators, and other adverse trophic interactions, thereby protecting any surviving (chaotrope‐tolerant) cells against damage and death from these biological threats. Therefore, it is to be expected that at moderate sublethal levels, the (water‐mediated) stress‐induced chaotropic salts can make cells of halophiles more resilient and tenacious, possibly enhancing their longevity. However, we have yet to determine whether chaotropic brines can under some circumstances preserve viable cells at salt concentrations above the known window for cellular metabolism. For example, up to what MgCl_2_ concentration can halophile cells be preserved over long timescales in an inactive‐yet‐viable condition; only up to 3 M or at > 3 M MgCl_2_?

## Life in vapour‐phase water

It is known that microbes can absorb and utilize water from the vapour phase (Rummel *et al*., 2014; Stevenson *et al*., 2015b). Notably, however, optimum longevity can occur at moderate‐to‐high relative humidity values; survival decreases for some microbes at low relative humidity values. In other words, at their ideal relative humidity and temperature combinations, some microbes can be preserved by vapour‐phase water.

For example, spores of the plant‐pathogenic basidiomycete *Phakopsora pachyrhizi* were stored at relative humidity values of 92.5%, 855%, 75%, 64%, and 47.5% (for one month) and germination assays carried out as a measure of survival, revealing that 92.5% and 85% relative humidity were the optimum values for preserving viability (Twizeyimana and Hartman, 2010). Daoust and Roberts (1983) stored conidia of the insect–pathogenic fungus *Metarhizium anisopliae* for a 2‐year period at a range of relative humidities (0, 12%, 33%, 53%, 75% and 98%). For those stored at physiological temperatures, 98% humidity preserved conidia most effectively (for those stored at 4°C, the highest viability was retained at 0% relative humidity, possibly due to cellular damage that can be caused by changes in the density of water at about 4°C, as discussed above). For another insect pathogen (*Beauveria bassiana*), a high relative humidity of 80% was used to preserve viability of spores over a period of > 2 years (Blanford *et al*., 2012). Similar results have been obtained for bacteria: *Bacillus subtilis* spores stored for 2 years at about 40% relative humidity did not exhibit any decrease in viability (in this study, higher humidities were not tested; Ulrich *et al*., 2018). In a separate study, cells of *Escherichia coli* stored at relative humidity ranges of 30–40%, 40–60%, and > 90% were better‐preserved at > 90% relative humidity than at lower values (Ng *et al*., 2017). Other examples for both bacteria and fungi are discussed in the review by Tang (2009).

Studies of microbial longevity at different relative humidities show that, as might be expected, survival depends on the type of microbe, its physiological condition, and the storage temperature used, so longevity experiments can yield various outcomes (Tang, 2009). Nevertheless, the findings highlighted here indicate that the effective concentration of vapour‐phase water molecules (relative humidity) can be critical to ensure survival under some circumstances. This can be critically important for microbes that inhabit surfaces (including those within soils and soil crusts; Lebre *et al*., 2017) and microbes present in the atmosphere, whether attached to particles, within aerosols, or ‘free’ (Archer *et al*., 2021). Such habitats are frequently dynamic; for example, there is an equilibrium between the vapour phase and condensed water. Condensation starts heterogeneously depending on the surface structure and occurs preferentially at microsites with capillary structure or electrical charges where the saturation vapour pressure is reduced (Mikhailov *et al*., 2004). Under some circumstances, condensation can immerse cells in liquid water more or less continuously. For example, a study of semi‐arid soil revealed that dew formation occurred over a daily window of 14 h and that the dew residence time was 18 h (Jia *et al*., 2019).

Water vapour, and aerosols, in Earth’s atmosphere are impacted by prevailing weather, altitude, latitude, anthropogenic activity, and volcanic activity (Robock, 2000; Satheesh and Krishnamoorthy, 2005; Hallsworth *et al*., 2021a). At a minimum, vapour‐phase water functions to maintain the cell membrane and other biomacromolecular structures and thereby preserve cellular integrity. In addition, moderate‐to‐high relative humidity mitigates against the high rates of oxidative damage that can occur during dehydration, is more favourable to basal‐yet‐essential metabolisms once absorbed by the cell (such as DNA repair; Bosch *et al*., 2021), and averts potentially lethal damage that can be caused by rapid rehydration that can follow desiccation (see below).

## Viable microbes in sugar‐rich aqueous milieux

For most honeys, the water activity (0.67 to 0.53) and acidity are so low (pH < 4) that no microorganisms, not even sugar‐adapted xerophiles, are metabolically active (Lievens *et al*., 2015). Therefore, microbes rarely spoil honey, and ancient honeys have been found that are up to 5500 years old (Lomsadze, 2012). Studies of modern honeys indicate that viable microbes can be well preserved, including pathogenic species (Abdulla *et al*., 2012; Grabowski and Klein, 2017). Preserved low‐moisture fruits and residues of ancient beers can also have a sufficiently low water‐activity to survive over long timescales. Sugar molecules have a substantial hydration shell and are hygroscopic, so microorganisms within high‐sugar materials remain immersed in/ hydrated by water. Whereas attempts have not always been made to culture microbes from ancient sugar‐rich materials—that are effectively highly concentrated aqueous solutions—viable microbes have been retrieved in some cases. Although sugars were not analysed, yeasts have been cultured from clay beer vessels that were 5000 years old (Aouizerat *et al*., 2019, 2020; Brüssow, 2020). Whereas Yashina *et al*. (2012) regenerated plants from 30 000‐year‐old fruits (and no attempt was made to culture viable microbes), these higher plants can survive within permafrost then it is likely that these high‐sugar fruits were associated with preserved microbes; life forms that are generally more simple and more robust.

## Other ways in which water preserves life

Water is clearly capable of facilitating the long‐term preservation of microbial cells. What is not yet resolved is the degree to which water preserves microbial life by mitigating against things such as biocidal factors. These may include, for example, reducing non‐ionising radiation; averting cell death due to trauma or senescence caused by recurrent stresses; minimizing cellular exposure to lethal chemicals or reactive oxygen species; providing for the structure and preservation of biomacromolecular systems; and rejuvenating and invigorating the cellular system.

### Water mitigates biocidal factors

Water can protect cells against potentially lethal phenomena as detailed below. Specifically, these biocidal factors are: (i) ultraviolet radiation; (ii) events which cause trauma to the cell membrane or could lead to death or the senescence or potential exhaustion of cells from recurrent desiccation–rehydration, freeze–thawing and extreme thermal shock (Morley *et al*., 1983; Moger‐Reischer and Lennon, 2019); (iii) high concentrations of chaotropes or other potentially lethal stressors (both solutes and hydrophobic molecules), and toxins/toxicants such as heavy metals, pesticide residues and metabolites, such as antibiotics, produced by competing microbes; and (iv) dehydration associated with increased oxidative damage.

Depending on the environment, prevailing conditions, and type of microbe and its physiological condition, each of factors i–iv could result in death. In some cases, cell death from these factors can be instantaneous (e.g. extreme cold or heat shock). In other cases (e.g. some solute stressors), death may result from the inability to generate energy and/or otherwise repair damage to the cell’s structural or physiological integrity. Each of these possibilities is discussed further below. As explained by Moger‐Reischer and Lennon (2019) in a discussion of microbial senescence: ‘Not all damage can be fixed owing to the time and energy costs of repairing the diverse types of damage that accrue in cells’.

#### i. Shielding from ultraviolet radiation

For microbes at the planet’s surface, ultraviolet radiation can kill cells via DNA and protein damage (Krisko and Radman, 2010). DNA is damaged due to direct interaction between DNA and UV‐B radiation, which leads to the formation of pyrimidine dimers between adjacent bases (Slieman and Nicholson, 2000; Cutler and Zimmerman, 2011). Proteins, DNA, and lipids are also damaged by reactive oxygen species, which are mostly produced by UV‐A radiation. While it was thought that DNA damage causes cell death, evidence from numerous studies now suggests that protein damage is a/the primary cause of lethality (Krisko and Radman, 2010; Radman, 2016). Without a healthy proteome, even DNA repair may be compromised; for as Radman (2016) observed, ‘a perfect genome is useless in the absence of active proteins’.

Global climate change involves ozone depletion and a corresponding increase in the ultraviolet radiation reaching Earth’s surface. Therefore, there has been a resurgence of research into the degree to which ultraviolet radiation can penetrate water; water reflects some ultraviolet radiation at the surface (typically less than 10%) and absorbs the remainder in proportion to depth in the water column (e.g. Houskeeper *et al*., 2021). Ultraviolet radiation is sharply reduced with depth in a water column by dissolved organic compounds such as humic and fulvic acids, absorbance and scattering by particulates, and absorbance by water molecules themselves. By contrast, ultraviolet radiation is not strongly absorbed by dissolved salts such as NaCl and MgCl_2_ (Godin *et al*., 2020). Ultraviolet radiation with energies less than ~ 10 eV can cause electronic excitation resulting in O‐H bond cleavage of water, but ultraviolet photons with energies above ~ 10 eV may ionize water, leading to reactive species (Arumainayagam *et al*., 2019). This reduction in ultraviolet penetration is described by an exponential decay equation which includes a wavelength‐dependent attenuation coefficient (in m^−1^), the intensity of light reaching the surface, and depth of the water column (in m) (Rose *et al*., 2019).

In general, UV‐B radiation is absorbed more strongly than UV‐A radiation. For waters containing organics and/or particulates, 90% of ultraviolet radiation is absorbed within a few centimetres from the surface (Tedetti and Sempéré, 2006; Hooker *et al*., 2013; Rose *et al*., 2019; Houskeeper *et al*., 2021). In waters which lack significant levels of dissolved organics or particulates (such as the open ocean), 90% UV‐A radiation and UV‐B radiation are absorbed at depths ranging from 20 to 56 m and 5 to 18 m, respectively (Tedetti and Sempéré, 2006; Overmans and Agustí, 2019). Accordingly, some microbial cells and communities correlate inversely with ultraviolet radiation down through the water column in relation to ecophysiology and longevity/survival (Avila‐Alonso *et al*., 2017; García‐Corral *et al*., 2020a,b).

Water in other physical states is also effective at shielding organisms from radiation insults. Snow and ice can reduce ultraviolet radiation more strongly than liquid water, through a mixture of scattering, reflection and absorption (Warren *et al*., 2006). For instance, a 5‐cm snow covering containing ice layers and air bubbles can reduce ultraviolet irradiance by an order of magnitude (Cockell *et al*., 2002). Just millimetres of snow‐encrusted ice coverings can reduce ultraviolet irradiance by half (Cockell *et al*., 2002). This property of water ice has been used to define the ultraviolet‐protected areas on the Martian polar caps that are potentially habitable by some terrestrial microbes and where visible radiation can still reach (Córdoba‐Jabonero *et al*., 2005). Water vapour is also very effective at filtering ultraviolet (see below).

#### ii. Preventing desiccation–rehydration, freeze–thawing and extreme cold‐ or heat‐shock events

For cells that remain in liquid water, seawater, or brine for long time periods, potentially lethal desiccation–rehydration events (which is particularly dangerous if rapid or instantaneous) and freeze–thawing events are avoided. Even in frozen environments, cryopegs, brine channels and thin films can function as refuges where microorganisms are preserved. In other habitats, such as soils where traumatic events can cause severe stresses to individual cells and selective death of microbes within the community, there can be a major energetic cost to most microbes, consequences for composition of the microbiome, and reductions in ecosystem‐level functions such as nutrient cycling, trophic interactions and overall productivity (Schimel *et al*., 2007; Cray *et al*., 2013a). Many kinds of aqueous habitat can act as a highly effective buffer against such eventualities by mitigating against biocidal events at scales spanning from the individual cell to the entire ecosystem.

The specific gravity (relative density) of water is low, with a value of 1 (compared with 2.17 for NaCl and 7.87 for iron, for example) and its specific heat capacity is high. In the liquid phase, water therefore functions as a strong insulator against sudden changes in temperature and so can mitigate against trauma to the cell membrane that would result from a sudden decrease in the state of order of the bilayer. For example, extreme heat shock is lethal to microbial cells, by entropically disordering the cell membrane and/or triggering oxidative damage (Davidson and Schiestl, 2001; Tereshina *et al*., 2010). Similarly, cold shock can be lethal for some cells (Drobnis *et al*., 1993; Cao‐Hoang *et al*., 2008). By insulating against extreme thermal changes, bodies of water can help to maintain the thermodynamic stability of the cell’s macromolecules, the extracellular milieu and the entire habitat. In hostile environments (those that are extremely cold, have a low water‐activity that prevents metabolic processes, and/or lack sources of nutrients), water’s property of buffering against extremes thereby minimizes the cell’s need for energy.

#### iii. Dilution or expulsion of stressors, toxins and toxicants

Microbial habitats can contain lethal concentrations of natural and anthropogenic solutes and hydrophobic compounds. These include chaotropic salts such as MgCl_2_; soluble and hydrophobic compounds released from microbes including antibiotics and other antimicrobial metabolites (Cray *et al*., 2013a); toxic products of metabolism including breakdown products of saprotrophic degradation (Cray *et al*., 2015); components of crude oil; heavy metals; and pesticide residues. The action of water (and seawater or brine) can under many circumstances reduce the concentration and bioavailability of such substances, at various scales, by dilution or by ‘expelling’ them from the aqueous phase so that they remain at sublethal levels.

This occurs when, for example, inhibitory metabolites become diluted in the aqueous milieu outside the cell membrane or volatilize (i.e. they are effectively purged from the aqueous phase by the polarity of water, due to their hydrophobicity); when pollutants are leached from soil into bulk water; or when there is ingress of water into the cell’s environment via surface runoff, precipitation, or tides (Aliste *et al*., 2020; Grimene *et al*., 2021). It may seem self‐evident that water can preserve life by diluting these lethal substances, yet this property is so manifestly obvious that it can easily be overlooked. Examples are given above showing that water can dilute chaotropic salts (e.g. Dead Sea brine) or other potentially lethal cocktails, such as acid brines of Western Australia that are highly osmotically stressful, highly acidic and high in heavy metals.

For water‐soluble organic compounds that are less polar than water, and hydrophobic compounds (log *P*_octanol–water_ ≥ 1.95; Bhaganna *et al*., 2010), the ‘expulsion’ principle is illustrated by the process used for high‐temperature production of bioethanol by microbial thermophiles at 45 to 72°C (Olson *et al*., 2015). Microbial inhibition due to chaotropicity and high temperature results largely from the entropic disordering of biomacromolecular systems, so these two stress parameters become even more potent when acting in concert (Cray *et al*., 2015). However, the effective aqueous fraction of ethanol is reduced at elevated temperatures in the range 45 to 72°C so water effectively ‘purges’ this alcohol from solution. This happens because the energy of the system means for a given temperature ethanol molecules escape the surface liquid (overcome the intermolecular forces) more readily than do those of the water.

Most cellular stressors, toxins and toxicants are less polar (have a higher log *P*_octanol–water_) than ethanol; see figure 1 of Bhaganna *et al*. (2010); tables 2 and 6 of Cray *et al*. (2013a); table 1 of Cray *et al*. (2013b); and figure 3 of Cray *et al*. (2015). Therefore, these substances have limited solubility and for the most part their modes‐of‐inhibitory action against the cell also relate to their chaotropicity or hydrophobicity (Cray *et al*., 2013a, 2015). Water limits both solubility and bioavailability for most of these potentially lethal stressors and toxins/toxicants (Bhaganna *et al*., 2010). Furthermore, the same phenomenon as that described above for bioethanol also occurs for many of these substances, and does so at much lower temperatures.

The same phenomenon occurs for those cellular stressors within crude oil, and many types of pesticide residues, which are typically hydrophobic so their miscibility limit and/or aqueous solubility are very low. For example, benzene (which is the most‐soluble substance within crude oil) has an aqueous solubility of about 25 mM at physiological temperatures, so excess benzene is ‘purged’ from solution (either into the vapour phase or a separate liquid phase); this phase separation means that cells in water are effectively protected from the excess benzene. Microorganisms such as some strains of *Pseudomonas* and some oil‐degrading microbes are able to retain minimal levels of metabolic activity and their capacity for cell division at 20–25 mM benzene. Microbes resistant to the cellular stress induced by such hydrophobic compounds are prevented from exposure to higher concentrations due to this water‐mediated expulsion of the hydrophobe from the system (Bhaganna *et al*., 2010).

#### iv. Averting damage from cellular dehydration

Cells in a state of extreme dehydration, or those which are desiccated, suffer from high levels of oxidative damage (Potts, 1994). Whereas repair processes may remain active in some such cells, the risk of lethal damage remains (Potts, 1994; Chang *et al*., 2020; Bosch *et al*., 2021). This can even occur in organisms that are well adapted to anhydrobiosis, but may do so to a lesser extent (Rapoport *et al*., 2019). Therefore, whereas Point ii (above) likely applies for some cells/conditions, extreme dehydration or desiccation is unlikely to favour longevity for most microorganisms. Phylogenetically diverse types of microorganisms are adapted for long‐term survival in an anhydrobiotic condition (Potts, 1994; Rapoport *et al*., 1995; Alpert, 2006; Ranawat and Rawat, 2017; Rapoport, 2019; Bosch *et al*., 2021), but for others (and even some of the former) it may be that long‐term preservation in some form of water (liquid water, vapour‐phase water, seawater, brine) is more favourable. For some cells, under some circumstances, it may be that the less molecular motion within a cell (during desiccation or long‐term freezing), the more effectively the cell is preserved. However, evidence presented in the current article suggests that in other cases the less change to a hydrated cell that is immersed in an aqueous liquid, the better the chances of survival.

### Stabilizing activity of ‘bound’ water

From the viewpoint of cellular function, desiccated cells by definition do not have liquid water/cytosol. Importantly, however, some water is retained even in lyophilized cells; this is historically termed ‘residual’ or ‘bound’ water, although studies show that it is not bound *per se* (Potts, 1994). In this way, desiccated cells are in fact partially hydrated and this water contributes to their preservation (Potts, 1994; Bosch *et al*., 2021). Some of the seminal studies on the biology of bound water were carried out in plant seeds (Vertucci, 1989, 1990). Subsequent studies, such as those of enzymes and cellular biology of *Saccharomyces cerevisiae* (Rapoport *et al*., 1995; Arjunan *et al*., 1996; Sano *et al*., 1999; Chen and Liao, 2002; Padmavathy *et al*., 2003; Bosch *et al*., 2021), focused on the microbiological aspects of bound water.

The residual water in low‐moisture lipid bilayers, such as those of dehydrated or desiccated cells, plays a role in maintaining lipid–lipid bonding and can help to maintain essential hydrophobic interactions (Potts, 1994; Patel and Frank, 2006; Roark and Feller, 2008), lipid–lipid proximity upon rehydration, and as a consequence, van der Waals attraction between adjacent lipids. Unfavourable entropic interactions between hydrophobic alkyl chains on lipids are the driving force for self‐aggregation of lipid molecules. However, the self‐organized structure of these lipids into the bilayer configuration critical for cell membrane integrity is dependent on the amount of water and the presence of other molecules, such as anions, cations and solvents, as well as temperature and pressure in the system (Dymond *et al*., 2016; Burrell *et al*., 2017). Water therefore appears to play a role in preserving the bilayer configuration and structural integrity and fluidity/energetics of the cell membrane (Dymond, 2016); and a structurally intact and functionally active membrane is critical to preservation of the cell (Crowe *et al*.,^1984^; Potts, 1994; Sano *et al*., 1999; Faria *et al*., 2009).

The concepts of residual or bound water(s) include monomolecular aqueous films and water molecules that hydrate, or form part of the secondary or tertiary structures of, organic macromolecules. The DNA helix, for example, is dependent on water for its structure (Ball, 2008). Similarly, proteins rely on bound water molecules for their structural–functional integrity (for a detailed discussion, see Ball, 2008). Indeed, desiccation by itself can trigger the denaturation of some proteins (Carpenter *et al*., 1993; Pikal‐Cleland and Carpenter, 2001). Whereas the bound water of cells is defined/determined with reference to the desiccated state and has been well‐studied (Crowe *et al*.,^1984^; Kanô *et al*., 1994; Tiwari and Tripathi, 1998; Ball, 2008; Dupont *et al*., 2014; Bosch *et al*., 2021), bound water also plays essential roles in the hydration and stability of biomacromolecules, maintains the integrity of the cell in its physiological state and facilitates protein functionality (Webb, 1965; Potts, 1994; Ball, 2008; Nomura *et al*., 2018; Murthy, 2021).

### Water‐mediated events may sometimes prolong life

For microbial cells which are resistant to cell membrane damage caused by freeze–thawing, desiccation followed by sudden rehydration, or thermal shock, it is plausible that recurrent bouts of stress imposed by such events can actually boost their vitality. Moderate levels of cellular stress can increase the cell’s generation of energy and stimulate damage‐repair mechanisms and other stress responses and adaptations (Hallsworth, 2018) so are also likely to increase the capacity for self‐repair for cells preserved long term in hostile environments (Moger‐Reischer and Lennon, 2019; Wright *et al*., 2020). It is therefore reasonable to hypothesize that, for some—maybe even most—microbes, repeated stressful events can (under the right circumstances) enhance their longevity. For example, within a microbial population or community, some cells will not survive these events; therefore, nutrients released from dead cells can be used by surviving cells (Schimel *et al*., 2007; Shoemaker *et al*., 2021). Evolutionary biology studies of soil microbes exposed to repeated cycles of freeze–thawing confirm adaptations to these events, albeit at the population level (Walker *et al*., 2006; Rosinger and Bonkowski, 2021). It is also plausible that individual cells have also adapted during these studies but further work would be needed to confirm this ‘stress‐induced cellular invigoration’ hypothesis.

## Water: A glass half empty or glass half full?

The fragility of cellular life is to some degree a product of its aqueous nature. Non‐covalent interactions that collectively bind the cell membrane, and enable its vital functions, also make it especially vulnerable to damage. The need for cell turgor makes life susceptible to osmotic shock/osmotic extremes and the high water content of cells and hydrated biomacromolecules can facilitate damage (or even death) by factors such as ionizing radiation (Nikitaki *et al*., 2016; Hall and Giaccia, 2018), heat shock/high temperature, desiccation–rehydration, freeze–thawing and chaotropic and hydrophobic substances (Hallsworth *et al*., 2003; Bhaganna *et al*., 2010).

Water also poses challenges for life at the level of physical chemistry. Speaking at a *Fitness of the Cosmos for Life* Workshop in 2003, Nobel Laureate and Harvard Professor Jack W. Szostak went so far as to say that water is ‘really a noxious, toxic, corrosive and generally lethal environment for life…[and] is probably the most genotoxic substance known’ (Supporting Information Appendix S2). He considers, for example, how hydrolytic reactions can destroy biomacromolecules (requiring, therefore, the evolution of energetically expensive repair systems); the instability of some metabolites in water; and the need for cellular membranes due to the strong solvent power of water (Supporting Information Appendix S2).

Despite these difficulties, life has evolved to become a fundamentally fluid, dynamic and ephemeral condition; a balancing act. Indeed, the very fabric of biology is made up of paradoxes. For instance, microbes utilize oxygen that is damaging, causing ageing and senescence (Dixon and Stockwell, 2014; Moger‐Reischer and Lennon, 2019); expend energy to counter the entropic degradation of cellular systems or the entire organism; evolve to adapt to their abiotic and biotic environment that in many cases undergoes constant change; and sustain damage (and may even begin to die) from the moment they first appear. It is, therefore, consistent with the nature of life that water provides both drawbacks and benefits.

## Mode(s) of action of water as a preservative

It would be difficult to mathematically model, or experimentally test, the hypothesis that water is a preservative of cellular life (e.g. what could be used as a meaningful control?). However, in contrast to organic solvents such as ethanol and formaldehyde that kill and preserve organisms (Grigorev and Korzhevskii, 2018), water is clearly a ‘biophilic’ solvent (Ball, 2008). Terrestrial life, coined ‘life as we know it’ by astrobiologists, evolved within the confines of the physical chemistry of water. It could be argued, therefore, that identifying water as a preservative is little more than an exercise in semantics. However, it is irrefutable that water is the milieu in which cells can remain stable, intact and alive over timescales measured not only in decades, but possibly many millions of years.

Furthermore, it is beyond doubt that diverse mechanisms are at play whereby water preserves cells in a living condition. In summary, these include:

- facilitating the secondary, tertiary and quaternary structures of biomacromolecular systems;
- buffering against thermodynamic extremes, partly due to water’s low specific gravity and high specific heat capacity;
- preventing traumatic events that could otherwise rupture the cell membrane (Calcott and MacLeod, 1975a,b; Kruuv *et al*., 1978; Crowe *et al*., 1984; Tanghe *et al*., 2004);
- mitigating against biocidal factors, not the least by reducing ultraviolet radiation and concentrations or bioavailability of solute stressors, hydrophobic stressors, and toxins/toxicants;
- preventing dehydration that increases oxidative damage;
- providing for the long‐term stability of the cell membrane under some circumstances;
- mediating moderate cellular stresses that can invigorate and rejuvenate cells; and
- providing strong electrostatic screening that allows highly charged ions and polyelectrolytes to remain soluble.

Water that is salt‐saturated acts to precipitate or redissolve salts as temperature varies, and thereby maintains a relatively consistent water activity even with moderate changes in temperature (Winston and Bates, 1960). This phenomenon helps to maintain a thermodynamically stable environment for adapted cells and thereby contributes to the habitability/survivability of some brines both under conditions that allow metabolic function (Lee *et al*., 2018) and those that do not.

The existence of two phase‐state changes—one at 0°C and one at 100°C—acts as a buffer for some microbial habitats. When there is a substantial body of liquid water exposed to large thermal changes, temperatures can be prevented from freezing or rising in temperature any higher. For instance, a cooling freshwater lake that is sufficiently large will not freeze, even at temperatures far below the 0°C freezing point of water. In case of a sustained ambient temperature drop below 0°C, the top layer will freeze in a floating layer of ice, which in turn creates a greenhouse effect beneath. In addition, once the water reaches 4°C, its increased density makes it sink which helps to maintain liquid water at depth where microorganisms avoid becoming frozen (Garrett *et al*., 2010; Bertilsson *et al*., 2013; Vollmer, 2019). Conversely, water or an aqueous solution exposed to high temperatures will release water vapour while maintaining the liquid temperature close to 100°C (depending on the solutes), thus preventing the water body or solution from rising to higher temperatures. Many types of microbial cells/communities can be preserved in liquid water at subzero temperatures, and some even around 100°C, so the capacity of water to act as a buffer against freeze–thawing events and temperatures of > 100°C contributes to preservation of cellular life for some microbes.

Water, being a fluid, also facilitates the removal of toxic substances from cells as discussed in the classic essay *The Fitness of the Environment* (Henderson, 1913). The roles that water plays in active biochemistry and the roles specific to warding off threats to the integrity of biomolecules and cells are not necessarily mutually exclusive. The ability of water to mediate medium range, relatively weak interactions—such as hydrophobic forces—is crucial in both cases. That it can support fast proton transfer by Grotthuss‐type hopping (whereby excess protons can move through a hydrogen‐bonded network of water molecules) is also important in many biological processes, and individual water molecules commonly function as ephemeral and mobile extensions to biomolecular structure by mediating protein–protein and protein–ligand recognition, for example (Ball, 2008).

In relation to water as a preservative, assuming the components of the cell membrane are chemically stable, or at least repairable, and the environment is fairly constant (temperature, pressure, etc.), then water molecules are apparently capable of maintaining membrane geometries over extended periods, possibly even geological timescales provided, of course, that these configurations represent thermodynamically stable, or at least kinetically trapped, states. In conclusion, water is required for a cell’s biochemical machinery to function (Ball, 2008) and without it, rather than just seizing up, life might often fall apart.

## Implications and perspectives

The preservation of foods is in large part based on depriving microbial cells of the water relations that would permit cell division/proliferation. This is achieved by increasing the concentration of low molecular‐weight solutes in the food(s); physically dehydrating, to reduce water activity; cooling or freezing (Qiu *et al*., 2019); and/or addition of preservative substances such as sodium benzoate, MgCl_2_ or ethanol that prevent microbial growth due to their chaotropicity (Alves *et al*., 2015), although this mechanism is generally not stated as such. Similarly, microbe‐mediated deterioration of books, documents, museum specimens, mummies, frescoes, paintings and other artefacts can be prevented by regulating the relative humidity and temperature regime (Arai, 2000; Cirigliano *et al*., 2021; He *et al*., 2021). These measures reflect the reality that H_2_O facilitates microbial activity that in turn drives decay.

Water is widely recognized for facilitating the structural–functional properties of cellular systems in addition to its role in promoting saprotrophic processes and biodeterioration. Indeed, water is often caricatured as an agent of decay (Chung *et al*., 2000; Brischke and Alfredsen, 2020; Fasuan *et al*., 2021). In the light of the evidence presented here, it is beyond doubt that, like anhydrobiosis (Dupont *et al*., 2014; Lebre *et al*., 2017; Bosch *et al*., 2021), immersion in water can facilitate long‐term preservation of cells. One hundred and fifty years ago, an authoritative (scientific and technical) English dictionary defined a preservative as: ‘That which…has the power of preserving; something that tends to secure a person or thing in a sound state, or prevent it from incurring injury, destruction, decay, or corruption… Persons formerly wore tablets of arsenic, as *preservatives* against the plague…Temperance and exercise are the best *preservatives* of health…’ (Ogilvie, 1868). So, whereas this term is used most commonly for substances that preserve foods, its application to the protection and conservation of microbes or other living systems should neither be viewed as contentious nor novel.

In relation to microbes located in salt deposits, McGenity *et al*. (2000) observed that ‘slow growth over geological time would be expected to decrease the mutation rate', alluding to the slowing down (or suspension) of microbial evolution in environments where microbes are inactive over immense timescales; a phenomenon also discussed by others (Maughan *et al*., 2002; Pedrós‐Alió, 2021). Water’s preservative activities also impact the ecologies and evolution of halophiles and other microorganisms by allowing them to circumvent hostile events or conditions (e.g. those preserved in a more or less inactive condition in oligotrophic or super‐cold water, permafrost, or hypersaline inclusions). Water thereby enables genomes that have been stored for immense time periods to be re‐released to resume life into what is essentially the present‐day (functional) biosphere, remote in time from the biosphere they once inhabited. Pedrós‐Alió (2021) recently published his paper: ‘Time travel in microorganisms’, that includes discussions pertinent to this phenomenon.

To some extent, we already utilize the preservative properties of water such as for the cryopreservation of viable microbes at subzero temperatures. It is possible, however, that re‐evaluating where, why, and how water preserves life in nature can inspire new biotechological applications. For example, to develop enhanced sterilization methodologies (Craven *et al*., 2021) or understand how and where unwanted human pathogens may persist in natural and built environments so that we can make knowledge‐based interventions (Cabral, 2010; Rhoads *et al*., 2015; Santos *et al*., 2015; Abioye *et al*., 2021; Vadell *et al*., 2021). In general, microorganisms are preserved in culture collections using –80°C storage or lyophilization (freeze‐drying). Only in limited cases (for some fungi, for example) is pure liquid water used for long‐term storage (Castro‐Rios and Bermeo‐Escobar, 2021). However, the preparation of such samples for storage is more time‐efficient, less expensive, and more environmentally sustainable so more studies ought to be carried out into the efficacy of water, seawater or brines to preserve other microbial structures/taxa at ambient temperatures. Importantly, storage in aqueous liquids also circumvents the potentially lethal cellular stresses associated with thawing or rapid rehydration.

Preparing the current article inspired the thought that live microbes able to provide health benefits (probiotics) might be added to frozen foods, but a literature search revealed that such work is already underway, for ice cream at least (Afzaal *et al*., 2020; Acu *et al*., 2021). Insights derived from studying preservative properties of water may also enable improvements of the hurdle technology (the methodology used to prevent the proliferation of pathogens in foods and animal feeds using stress parameters or other ‘hurdles’) (Qiu *et al*., 2019) by the systematic application of chaotropicity, for example. The principle that water repels apolar metabolites (many of which are inhibitory to microbes) might also be applied more widely to the biotechnological production of hydrophobic substances using thermophiles grown at high temperature.

### A comment on astrobiology

In the astrobiology field, it has become the norm to plan the search for biosignatures of past life by selecting places where water used to exist, such as the Jezero Crater of Mars that is currently being sampled by the Perseverance rover (Voosen, 2021), or is thought to currently exist, such as the oceans of Enceladus, Europa and Titan. However, more future space missions could also focus on waters themselves as a location where biosignatures exist. Intriguingly, for example, the Monaghans chondrite, partly composed of halite and thought to originate from early Mars, was found to have hypersaline fluid inclusions containing water that is 4 billion years old (see figure 2 of Zolensky *et al*., 2017). Given the efficacy of water as a long‐term preservative (and assuming that alien life is indeed water‐based), such ‘biosignatures’ may even take the form of extant life. This could have implications on planetary protection procedures: care ought to be taken to consider long‐term preservation of cells in aqueous milieu to avoid contamination of Mars or other planetary bodies during space exploration (Changela *et al*., 2021).

In a seminal study relating to potential origins of life on Earth and other planets, Bethell and Bergin (2009) used modelling approaches to reveal that water vapour shields both water and organics from ultraviolet and other forms of non‐ionizing radiation: ‘Similar to the ozone layer that protects Earth’s surface from the destructive effects of solar ultraviolet radiation, water created *in situ* at the…surface [of the rotating protoplanetary disc of gas and dust around a newly formed star] within a few astronomical units of the star will protect any water vapour either created via gas‐phase reactions or supplied to the midplane via evaporating icy planetesimals [debris likely to come together under gravitational forces to form a planet]. In addition, the surface water will protect any molecules created by gas‐phase chemistry, allowing for a rich organic chemistry to persist in the inner few astronomical units, even as the dust grains evolve toward planets’. Astrobiological lines of inquiry, therefore, may also provide insights pertinent to knowledge‐based terrestrial applications (Hallsworth *et al*., 2021b).

### Biosphere changes caused by global climate change

The terrestrial biosphere is changing drastically, driven by civil engineering projects, changes in land use, increasing human population, and the multifarious effects of climate change (Blois *et al*., 2013; Cavicchioli *et al*., 2019; Timmis and Hallsworth, 2022). Whereas the current (Anthropocene) mass extinction event is being monitored (Ceballos *et al*., 2020; Estes *et al.*, 2020), another major consequence is the release/reactivation of preserved microbes which were until now within the cryosphere (permafrost, the polar ice caps, glaciers and sea ice that have begun to melt); and the reactivation of near‐inactive microbes in hitherto nutrient‐depleted and cold freshwater, saline, and brine lakes and other places where change is beginning to occur. For example, permafrost is a repository for microbes of all domains of life (Kochkina *et al*., 2012; Sipes *et al*., 2021) and yet the increasing global temperatures and current forest fires in Siberia are thawing this massive ice deposit (Kirdyanov *et al*., 2020; Anisimov and Zimov, 2021).

The oceans, populated by microbes, are for the most part nutrient‐depleted (Kelly *et al*., 2021; Rech *et al*., 2021) so they also act as a planetary‐scale repository for viable microorganisms. It is likely that climate change will lead to the large‐scale release of nutrients into aqueous environments (Feng *et al*., 2020; Duerschlag *et al*., 2021; Kluge *et al*., 2021), lead to the emergence of new or old pathogens (Revich *et al*., 2012), and in places may promote the dissolution of some ancient salt deposits in which preserved microbes (from past biospheres) are abundant (Ramos‐Barbero *et al.*, 2021; Pedrós‐Alió, 2021). Episodes of climate change have occurred throughout the Earth’s geological history, driven by factors such as volcanic activity (Robock, 2000). The current (anthropogenic) episode of climate change, primarily hostile in its effect on life, will likely activate preserved microbes on an unprecedented scale. Indeed, this process has already begun, and will increase in the coming years as global temperatures increase (Tollefson, 2020). We have yet to evaluate future impacts on the ecology and evolutionary biology of microorganisms within the functional biosphere. It is, however, clear that the biology of water transcends various scales in space and time, and is intimately involved in the transitions that planet Earth is undergoing.

## Conflict of interest

None declared.

## Supporting information


**Appendix S1**. Dating of fluid inclusions of halite.

**Appendix S2**. Supporting Information (Szostak, 2003)

## Endnote

^1^
There have been occasional suggestions of active microbial life in saturated, MgCl_2_‐dominated brines (4.4 to 5 MgCl_2_, van der Wielen *et al*., 2005; Steinle *et al*., 2018); however, these were not based on observations or measurements of cellular biology.

## References

[mbt213980-bib-0001] Abdulla, C.O. , Ayubi, A. , Zulfiquer, F. , Santhanam, G. , Ahmed, M.A. , and Deeb, J. (2012) Infant botulism following honey ingestion. BMJ Case Rep 2012: bcr1120115153. 10.1136/bcr.11.2011.5153 PMC3448763 22962382

[mbt213980-bib-0002] Abioye, O.E. , Osunla, A.C. , and Okoh, A.I. (2021) Molecular detection and distribution of six medically important *Vibrio* spp. in selected freshwater and brackish water resources in Eastern Cape Province, South Africa. Front Microbiol 12: 617703. 34149632 10.3389/fmicb.2021.617703 PMC8208477

[mbt213980-bib-0003] Acu, M. , Kinik, O. , and Yerlikaya, O. (2021) Probiotic viability, viscosity, hardness properties and sensorial quality of synbiotic ice creams produced from goat’s milk. Food Sci Technol 41: 167–173.

[mbt213980-bib-0004] Afouda, P. , Dubourg, G. , and Raoult, D. (2020) Archeomicrobiology applied to environmental samples. Microb Pathog 143: 104140. 32173498 10.1016/j.micpath.2020.104140

[mbt213980-bib-0005] Afzaal, M. , Khan, A.U. , Saeed, F. , Arshad, M.S. , Khan, M.A. , Saeed, M. , *et al*. (2020) Survival and stability of free and encapsulated probiotic bacteria under simulated gastrointestinal conditions and in ice cream. Food Sci Nutr 8: 1649–1656. 32180972 10.1002/fsn3.1451 PMC7063362

[mbt213980-bib-0006] Aliste, M. , Pérez‐Lucas, G. , Vela, N. , Garrido, I. , Fenoll, J. , and Navarro, S. (2020) Solar‐driven photocatalytic treatment as sustainable strategy to remove pesticide residues from leaching water. Environ Sci Pollut Res Int 27: 7222–7233. 31883076 10.1007/s11356-019-07061-2

[mbt213980-bib-0007] Alpert, P. (2006) Constraints of tolerance: why are desiccation‐tolerant organisms so small or rare? J Exp Biol 209: 1575–1584. 16621938 10.1242/jeb.02179

[mbt213980-bib-0008] Andersen, D.T. , Sumner, D.Y. , Hawes, I. , Webster‐Brown, J. , and McKay, C.P. (2011) Discovery of large conical stromatolites in Lake Untersee, Antarctica. Geobiology 9: 280–293. 21504538 10.1111/j.1472-4669.2011.00279.x

[mbt213980-bib-0009] Anisimov, O. , and Zimov, S. (2021) Thawing permafrost and methane emission in Siberia: synthesis of observations, reanalysis, and predictive modeling. Ambio 50: 2050–2059. 33140207 10.1007/s13280-020-01392-y PMC8497670

[mbt213980-bib-0010] Aouizerat, T. , Gutman, I. , Paz, Y. , Maeir, A.M. , Gadot, Y. , Gelman, D. , *et al*. (2019) Isolation and characterization of live yeast cells from ancient vessels as a tool in bio‐archaeology. MBio 10: e00388‐19. 31040238 10.1128/mBio.00388-19 PMC6495373

[mbt213980-bib-0011] Aouizerat, T. , Maeir, A.M. , Paz, Y. , Gadot, Y. , Szitenberg, A. , Alkalay‐Oren, S. , *et al*. (2020) Isolation and characterization of live yeast cells from ancient clay vessels. Bio Protoc 10: e3473. 10.21769/BioProtoc.3473 PMC7842698 33654708

[mbt213980-bib-0012] Arahal, D.R. , Gutiérrez, M.C. , Elazari‐Volcani, B. , and Ventosa, A. (2000) Taxonomic analysis of extremely halophilic archaea isolated from 56‐years‐old Dead Sea brine samples. Syst Appl Microbiol 23: 376–385. 11108017 10.1016/S0723-2020(00)80068-5

[mbt213980-bib-0013] Arai, H. (2000) Foxing caused by Fungi: twenty‐five years of study. Int Biodeter Biodegr 46: 181–188.

[mbt213980-bib-0014] Archer, S. , Lee, K. , Caruso, T. , Leung, M. , Tong, X. , Hinchliffe, G. , *et al*. (2021) Diverse recruitment to a globally structured atmospheric microbiome. Research Square. 10.21203/rs.3.rs-244923/v2.

[mbt213980-bib-0015] Arjunan, P. , Umland, T. , Dyda, F. , Swaminathan, S. , Furey, W. , Sax, M. , *et al*. (1996) Crystal structure of the thiamin diphosphate‐dependent enzyme pyruvate decarboxylase from the yeast *Saccharomyces cerevisiae* at 2.3 Å resolution. J Mol Biol 256: 590–600. 8604141 10.1006/jmbi.1996.0111

[mbt213980-bib-0016] Arumainayagam, C.R. , Garrod, R.T. , Boyer, M.C. , Hay, A.K. , Bao, S.T. , Campbell, J.S. , *et al*. (2019) Extraterrestrial prebiotic molecules: photochemistry vs. radiation chemistry of interstellar ices. Chem Soc Rev 48: 2293–2314. 30815642 10.1039/c7cs00443e

[mbt213980-bib-0017] Avila‐Alonso, D. , Baetens, J.M. , Cardenas, R. , and De Baets, B. (2017) Modeling the effect of ultraviolet radiation on the photosynthetic potential of *Prochlorococcus* and *Synechococcus* cyanobacteria. Aquat Microb Ecol 79: 149–164.

[mbt213980-bib-0018] Bakermans, C. (2017) Determining the limits of microbial life at subzero temperatures. In Psychrophiles: From Biodiversity to Biotechnology. Margesin, R. (ed). Berlin: Springer, pp. 21–38.

[mbt213980-bib-0019] Ball, P. (2008) Water as an active constituent in cell biology. Chem Rev 108: 74–108. 18095715 10.1021/cr068037a

[mbt213980-bib-0020] Ball, P. (2017) Water is an active matrix of life for cell and molecular biology. Proc Natl Acad Sci USA 114: 13327–13335. 28592654 10.1073/pnas.1703781114 PMC5754758

[mbt213980-bib-0021] Ball, P. , and Hallsworth, J.E. (2015) Water structure and chaotropicity: their uses, abuses and biological implications. Phys Chem Chem Phys 17: 8297–8305. 25628033 10.1039/c4cp04564e

[mbt213980-bib-0022] Belilla, J. , Moreira, D. , Jardillier, L. , Reboul, G. , Benzerara, K. , López‐García, J.M. , *et al*. (2019) Hyperdiverse archaea near life limits at the polyextreme geothermal Dallol area. Nat Ecol Evol 3: 1552–1561. 31666740 10.1038/s41559-019-1005-0 PMC6837875

[mbt213980-bib-0023] Benison, K.C. (2008) Life and death around acid‐saline lakes. Palaios 23: 571–573.

[mbt213980-bib-0024] Benison, K.C. (2019) How to search for life in Martian chemical sediments and their fluid and folid inclusions using petrographic and spectroscopic methods. Front Environ Sci 7: 1–15.

[mbt213980-bib-0025] Benison, K.C. , O’Neill, W.K. , Blain, D. , and Hallsworth, J.E. (2021) Water activities of acid brine lakes approach the limit for life. Astrobiology 21: 729–740. 33819431 10.1089/ast.2020.2334 PMC8219186

[mbt213980-bib-0026] Bertilsson, S. , Burgin, A. , Carey, C.C. , Fey, S.B. , Grossart, H.‐P. , Grubisic, L.M. , *et al*. (2013) The under‐ice microbiome of seasonally frozen lakes. Limnol Oceanogr 58: 1998–2002.

[mbt213980-bib-0027] Bethell, T. , and Bergin, E. (2009) Formation and survival of water vapor in the terrestrial planet‐forming region. Science 326: 1675–1677. 20019283 10.1126/science.1176879

[mbt213980-bib-0028] Bhaganna, P. , Volkers, R. J. M. , Bell, A. N. W. , Kluge, K. , Timson, D. J. , McGrath, J. W. , *et al*. (2010) Hydrophobic substances induce water stress in microbial cells. Microbial Biotechnol 3: 701–716. 10.1111/j.1751-7915.2010.00203.x PMC3815343 21255365

[mbt213980-bib-0029] Bidle, K.D. , Lee, S.H. , Marchant, D.R. , and Falkowski, P.G. (2007) Fossil genes and microbes in the oldest ice on Earth. Proc Natl Acad Sci USA 104: 13455–13460. 17686983 10.1073/pnas.0702196104 PMC1941643

[mbt213980-bib-0030] Bird, J.T. , Tague, E.D. , Zinke, L. , Schmidt, J.M. , Steen, A.D. , Reese, B. , *et al*. (2019) Uncultured microbial phyla suggest mechanisms for multi‐thousand‐year subsistence in Baltic sea sediments. MBio 10: e02376‐18. 10.1128/mBio.02376-18 PMC6469976 30992358

[mbt213980-bib-0031] Blanford, S. , Jenkins, N.E. , Christian, R. , Chan, B.H.K. , Nardini, L. , Osae, M. , *et al*. (2012) Storage and persistence of a candidate fungal biopesticide for use against adult malaria vectors. Malar J 11: 354. 23098323 10.1186/1475-2875-11-354 PMC3506477

[mbt213980-bib-0032] Blois, J.L. , Zarnetske, P.L. , Fitzpatrick, M.C. , and Finnegan, S. (2013) Climate change and the past, present, and future of biotic interactions. Science 341: 499–504. 23908227 10.1126/science.1237184

[mbt213980-bib-0033] Bodaker, I. , Sharon, I. , Suzuki, M.T. , Feingersch, R. , Shmoish, M. , Andreishcheva, E. , *et al*. (2010) Comparative community genomics in the Dead Sea: an increasingly extreme environment. ISME J 4: 399–407. 20033072 10.1038/ismej.2009.141

[mbt213980-bib-0034] Bosch, J. , Varliero, G. , Hallsworth, J.E. , Dallas, T.D. , Hopkins, D. , Frey, B. , *et al*. (2021) Microbial anhydrobiosis. Environ Microbiol 23: 6377–6390. 34347349 10.1111/1462-2920.15699

[mbt213980-bib-0035] Brischke, C. , and Alfredsen, G. (2020) Wood‐water relationships and their role for wood susceptibility to fungal decay. Appl Microbiol Biotechnol 104: 3781–3795. 32144473 10.1007/s00253-020-10479-1 PMC8326242

[mbt213980-bib-0036] Brown, A.D. (1976) Microbial water stress. Bacteriol Rev 40: 803–846. 1008746 10.1128/br.40.4.803-846.1976 PMC413986

[mbt213980-bib-0037] Brüssow, H. (2020) Bioarchaeology: a profitable dialogue between microbiology and archaeology. Microb Biotechnol 13: 406–409. 32053292 10.1111/1751-7915.13527 PMC7017812

[mbt213980-bib-0038] Bui, V.T. , Ahad, E. , Rheaume, D. , and Whitehead, R. (1997) Evaluation of branched glycidyl azide polymer purified by solvent extraction. Ind Eng Chem Res 36: 2219–2224.

[mbt213980-bib-0039] Bulat, S.A. (2016) Microbiology of the subglacial Lake Vostok: first results of borehole‐frozen lake water analysis and prospects for searching for lake inhabitants. Philos Trans A Math Phys Eng Sci 374: 20140292. 26667905 10.1098/rsta.2014.0292

[mbt213980-bib-0040] Buongiorno, J. , Turner, S. , Webster, G. , Asai, M. , Shumaker, A.K. , Roy, T. , *et al*. (2017) Interlaboratory quantification of Bacteria and Archaea in deeply buried sediments of the Baltic Sea (IODP Expedition 347). FEMS Microbiol Ecol 93: fix007. 10.1093/femsec/fix007 28104666

[mbt213980-bib-0041] Burdsall, H.H. , and Dorworth, E.B. (1994) Preserving cultures of wood‐decaying Basidiomycotina using sterile distilled water in cryocials. Mycologia 86: 275–280.

[mbt213980-bib-0042] Burrell, J. , Dymond, M.K. , Gillams, R.J. , Parker, D.J. , Langley, G.J. , *et al*. (2017) Using curvature power to map the domain of inverse micellar cubic phases: The case of aliphatic aldehydes in 1,2‐dioleoyl‐*sn*‐glycero‐3‐phosphoethanolamine. Langmuir 33: 12804–12813. 28981289 10.1021/acs.langmuir.7b02998

[mbt213980-bib-0043] Cabral, J.P. (2010) Water microbiology. Bacterial pathogens and water. Int J Environ Res Public Health 7: 3657–3703. 21139855 10.3390/ijerph7103657 PMC2996186

[mbt213980-bib-0044] Calcott, P.H. , and MacLeod, R.A. (1975a) The survival of *Escherichia coli* from freeze‐thaw damage: permeability barrier damage and viability. Can J Microbiol 21: 1724–1732. 1104119 10.1139/m75-253

[mbt213980-bib-0045] Calcott, P.H. , and MacLeod, R.A. (1975b) The survival of *Escherichia coli* from freeze‐thaw damage: the relative importance of wall and membrane damage. Can J Microbiol 21: 1960–1968. 766928 10.1139/m75-284

[mbt213980-bib-0046] Cao‐Hoang, L. , Dumont, F. , Marechal, P.A. , Le‐Thanh, M. , and Gervais, P. (2008) Rates of chilling to 0°C: Implications for the survival of microorganisms and relationship with membrane fluidity modifications. Appl Microbiol Biotechnol 77: 1379–1387. 18060401 10.1007/s00253-007-1279-z

[mbt213980-bib-0047] Carpenter, J.F. , Prestrelski, S.J. , and Arakawa, T. (1993) Separation of freezing‐ and drying‐induced denaturation of lyophilized proteins using stress‐specific stabilization. I. Enzyme activity and calorimetric studies. Arch Biochem Biophys 303: 456–464. 8512328 10.1006/abbi.1993.1309

[mbt213980-bib-0048] Castro‐Rios, K. , and Bermeo‐Escobar, L.P. (2021) Methods for the culture conservation of edible and medicinal fungi. J Microbiol Biotechnol Food Sci 10: 620–625.

[mbt213980-bib-0049] Cavicchioli, R. , Ripple, W.J. , Timmis, K.N. , Azam, F. , Bakken, L.R. , Baylis, M. , *et al*. (2019) Scientists’ warning to humanity: microorganisms and climate change. Nat Rev Microbiol 17: 569–586. 31213707 10.1038/s41579-019-0222-5 PMC7136171

[mbt213980-bib-0050] Ceballos, G. , Ehrlich, P.R. , and Raven, P.H. (2020) Vertebrates on the brink as indicators of biological annihilation and the sixth mass extinction. Proc Natl Acad Sci USA 117: 13596–13602. 32482862 10.1073/pnas.1922686117 PMC7306750

[mbt213980-bib-0051] Chan, Q.H.S. , Zolensky, M.E. , Kebukawa, Y. , Fries, M. , Ito, M. , Steele, A. , *et al*. (2018) Organic matter in extraterrestrial water‐bearing salt crystals. Sci Adv 4: eaao3521. 29349297 10.1126/sciadv.aao3521 PMC5770164

[mbt213980-bib-0052] Chang, R.L. , Stanley, J.A. , Robinson, M.C. , Sher, J.W. , Li, Z. , Chan, Y.A. , *et al*. (2020) Protein structure, amino acid composition and sequence determine proteome vulnerability to oxidation‐induced damage. EMBO J 39: e104523. 33073387 10.15252/embj.2020104523 PMC7705453

[mbt213980-bib-0053] Changela, H.G. , Chatzitheodoridis, E. , Anuntes, A. , Beaty, D. , Bouw, K. , Bridges, J.C. , *et al*. (2021) Mars: new insights and unresolved questions. Internat J Astrobiol 20: 394–426.

[mbt213980-bib-0054] Chen, D.H. , and Liao, M.H. (2002) Effects of mixed reverse micellar structure on stability and activity of yeast alcohol dehydrogenase. J Mol Catal B 18: 155–162.

[mbt213980-bib-0055] Christner, B.C. , Mosley‐Thompson, E. , Thompson, L.G. , Zagorodnov, V. , Sandman, K. , and Reeve, J.N. (1999) Recovery and identification of viable bacteria immured in glacial ice. Icarus 144: 479–485.

[mbt213980-bib-0056] Christner, B.C. , Mosley‐Thompson, E. , Thompson, L.G. , and Reeve, J.N. (2003) Bacterial recovery from ancient glacial ice. Environ Microbiol 5: 433–436. 12713469 10.1046/j.1462-2920.2003.00422.x

[mbt213980-bib-0057] Chung, Y.C. , Chen, H.C. , Shyu, Y.T. , and Hua, J. (2000) Temperature and water effects on the biodeterioration for marine fuel oil. Fuel 79: 1525–1532.

[mbt213980-bib-0058] Cirigliano, A. , Mura, F. , Cecchini, A. , Tomassetti, M.C. , Maras, D.F. , Di Paola, M. , *et al*. (2021) Active microbial ecosystem in Iron‐Age tombs of the Etruscan civilization. Environ Microbiol 23: 3957–3969. 33200556 10.1111/1462-2920.15327

[mbt213980-bib-0059] Clarke, A. (2014) The thermal limits to life on Earth. Int J Astrobiol 13: 141–154.

[mbt213980-bib-0060] Clarke, A. , Morris, G.J. , Fonseca, F. , Murray, B.J. , Acton, E. , and Price, H.C. (2013) A low temperature limit for life on Earth. PLoS One 8: e66207. 23840425 10.1371/journal.pone.0066207 PMC3686811

[mbt213980-bib-0061] Cockell, C.S. , Rettberg, P. , Horneck, G. , Wynn‐Williams, D.D. , Scherer, K. , and Gugg‐Helminger, A. (2002) Influence of ice and snow covers on the UV exposure of terrestrial microbial communities: dosimetric studies. J Photochem Photobiol B 68: 23–32. 12208033 10.1016/s1011-1344(02)00327-5

[mbt213980-bib-0062] Colangelo‐Lillis, J. , Eicken, H. , Carpenter, S.D. , and Deming, J.W. (2016) Evidence for marine origin and microbial‐viral habitability of sub‐zero hypersaline aqueous inclusions within permafrost near Barrow, Alaska. FEMS Microbiol Ecol 92: fiw053. 26976841 10.1093/femsec/fiw053

[mbt213980-bib-0063] Combarnous, Y. , and Nguyen, T.M.D. (2020) Cell communications among microorganisms, plants, and animals: Origin, evolution, and interplays. Int J Mol Sci 21: 8052. 10.3390/ijms21218052 PMC7663094 33126770

[mbt213980-bib-0064] Coolen, M.J.L. , and Orsi, W.D. (2015) The transcriptional response of microbial communities in thawing Alaskan permafrost soils. Front Microbiol 6: 197. 25852660 10.3389/fmicb.2015.00197 PMC4360760

[mbt213980-bib-0065] Cooper, Z.S. , Rapp, J.Z. , Carpenter, S.D. , Iwahana, G. , Eicken, H. , and Deming, J.W. (2019) Distinctive microbial communities in subzero hypersaline brines from Arctic coastal sea ice and rarely sampled cryopegs. FEMS Microbiol Ecol 95: fiz166. 10.1093/femsec/fiz166 PMC6859516 31626297

[mbt213980-bib-0066] Córdoba‐Jabonero, C. , Zorzano, M.P. , Selsis, F. , Patel, M.R. , and Cockell, C.S. (2005) Radiative habitable zones in Martian polar environments. Icarus 175: 360–371. 16044598 10.1016/j.icarus.2004.12.009

[mbt213980-bib-0067] Cragg, B.A. , Parkes, R.J. , Fry, J.C. , Weightman, A.J. , Rochelle, P.A. , and Maxwell, J.R. (1996) Bacterial populations and processes in sediments containing gas hydrates (ODP Leg 146: Cascadia Margin). Earth Planet Sci Lett 139: 497–507.

[mbt213980-bib-0068] Craven, E. , Winters, M. , Smith, A.L. , Lalime, E. , Mancinelli, R. , Shirey, B. , *et al*. (2021) Biological safety in the context of backward planetary protection and Mars Sample Return: conclusions from the Sterilization Working Group. Int J Astrobiol 20: 1–28.

[mbt213980-bib-0069] Cray, J.A. , Bell, A.N.W. , Bhaganna, P. , Mswaka, A.Y. , Timson, D.J. , and Hallsworth, J.E. (2013a) The biology of habitat dominance; can microbes behave as weeds? Microb Biotechnol 6: 453–492. 23336673 10.1111/1751-7915.12027 PMC3918151

[mbt213980-bib-0070] Cray, J.A. , Russell, J.T. , Timson, D.J. , Singhal, R.S. , and Hallsworth, J.E. (2013b) A universal measure of chaotropicity and kosmotropicity. Environ Microbiol 15: 287–296. 23145833 10.1111/1462-2920.12018

[mbt213980-bib-0071] Cray, J.A. , Stevenson, A. , Ball, P. , Bankar, S.B. , Eleutherio, E.C.A. , Ezeji, T.C. , *et al*. (2015) Chaotropicity: a key factor in product tolerance of biofuel‐producing microorganisms. Curr Opin Biotechnol 33: 258–259. 10.1016/j.copbio.2015.02.010 25841213

[mbt213980-bib-0072] Crowe, J.H. , Crowe, L.M. , and Chapman, D. (1984) Preservation of membranes in anhydrobiotic organisms: The role of trehalose. Science 17: 701–703. 10.1126/science.223.4637.701 17841031

[mbt213980-bib-0073] Cuiec, L.E. , Bourbiaux, B. , and Kalaydjian, F. (1994) Oil recovery by imbibition in low‐permeability chalk. SPE Form Eval 9: 200–208.

[mbt213980-bib-0074] Cunningham, J.A. , Liu, A.G. , Bengtson, S. , and Donoghue, P.C. (2017) The origin of animals: Can molecular clocks and the fossil record be reconciled? BioEssays 39: 1–12. 10.1002/bies.201600120 27918074

[mbt213980-bib-0075] Cutler, T.D. , and Zimmerman, J.J. (2011) Ultraviolet irradiation and the mechanisms underlying its inactivation of infectious agents. Anim Health Res Rev 12: 15–23. 21676338 10.1017/S1466252311000016

[mbt213980-bib-0076] Daffonchio, D. , Borin, S. , Brusa, T. , Brusetti, L. , van der Wielen, P.W.J.J. , Bolhuis, H. , *et al*. (2006) Stratified prokaryote network in the oxic–anoxic transition of a deep‐sea halocline. Nature 440: 203–207. 16525471 10.1038/nature04418

[mbt213980-bib-0077] Daniel, R.M. , Finney, J.L. , and Stoneham, M. (eds) (2004) The molecular basis of life: is life possible without water? Philos Trans R Soc Lond B Biol Sci 359: 1141–1328.

[mbt213980-bib-0078] Daoust, R.A. , and Roberts, D.W. (1983) Studies on the prolonged storage of *Metarhizium anisopliae* conidia: effect of temperature and relative humidity on conidial viability and virulence against mosquitoes. J Invertebr Pathol 41: 143–150. 6841994 10.1016/0022-2011(83)90213-6

[mbt213980-bib-0079] Davidson, J.F. , and Schiestl, R.H. (2001) Cytotoxic and genotoxic consequences of heat stress are dependent on the presence of oxygen in *Saccharomyces cerevisiae* . J Bacteriol 183: 4580–4587. 11443093 10.1128/JB.183.15.4580-4587.2001 PMC95353

[mbt213980-bib-0081] de Lima Alves, F. , Stevenson, A. , Baxter, E. , Gillion, J.L.M. , Hejazi, F. , Hayes, S. , *et al*. (2015) Concomitant osmotic and chaotropicity‐induced stresses in *Aspergillus wentii*: compatible solutes determine the biotic window. Curr Gen 61: 457–477. 10.1007/s00294-015-0496-8 26055444

[mbt213980-bib-0082] Dixon, S.J. , and Stockwell, B.R. (2014) The role of iron and reactive oxygen species in cell death. Nat Chem Biol 10: 9–17. 24346035 10.1038/nchembio.1416

[mbt213980-bib-0083] Dombrowski, H.J. (1966) Geological problems in the question of living bacteria in Paleozoic salt deposits. In Second Symposium on Salt, Vol. 1. Rau, J. L. (ed.). Cleveland, OH, USA: Northern Ohio Geological Society, pp. 215–219.

[mbt213980-bib-0084] Drobnis, E.Z. , Crowe, L.M. , Berger, T. , Anchordoguy, T.J. , Overstreet, J.W. , and Crowe, J.H. (1993) Cold shock damage is due to lipid phase transitions in cell membranes: a demonstration using sperm as a model. J Exp Zool 265: 432–437. 8463792 10.1002/jez.1402650413

[mbt213980-bib-0085] Duda, V.I. , Danilevich, V.N. , Suzina, N.E. , Shorokhova, A.P. , Dmitriev, V.V. , Mokhova, O.N. , and Akimov, V.N. (2004) Changes in the fine structure of microbial cells induced by chaotropic salts. Microbiology 73: 341–349. 15315236

[mbt213980-bib-0086] Duda, V.I. , Danilevich, V.N. , Akimov, V.N. , Suzina, N.E. , Dmitriev, V.V. , and Shorokhova, A.P. (2005) Fluorescence microscopic study of microorganisms treated with chaotropic agents. Microbiology 74: 434–439. 16211854

[mbt213980-bib-0087] Duerschlag, J. , Mohr, W. , Ferdelman, T.G. , LaRoche, J. , Desai, D. , Croot, P.L. , *et al*. (2021) Niche partitioning by photosynthetic plankton as a driver of CO_2_‐fixation across the oligotrophic South Pacific Subtropical Ocean. ISME J. 10.1038/s41396-021-01072-z. PMC8776750 34413475

[mbt213980-bib-0088] Dupont, S. , Rapoport, A. , Gervais, P. , and Beney, L. (2014) Survival kit of *Saccharomyces cerevisiae* for anhydrobiosis. Appl Microbiol Biotechnol 98: 8821–8834. 25172136 10.1007/s00253-014-6028-5

[mbt213980-bib-0089] Dymond, M.K. (2016) Mammalian phospholipid homeostasis: evidence that membrane curvature elastic stress drives homeoviscous adaptation *in vivo* . J R Soc Interface 13: 20160228. 27534697 10.1098/rsif.2016.0228 PMC5014058

[mbt213980-bib-0090] Dymond, M.K. , Gillams, R.J. , Parker, D.J. , Burrell, J. , Labrador, A. , Nylander, T. , and Attard, G.S. (2016) Lipid spontaneous curvatures estimated from temperature‐dependent changes in inverse hexagonal phase lattice parameters: effects of metal cations. Langmuir 32: 10083–10092. 27603198 10.1021/acs.langmuir.6b03098

[mbt213980-bib-0091] Elabed, H. , González‐Tortuero, E. , Ibacache‐Quiroga, C. , Bakhrouf, A. , Johnston, P. , Gaddour, K. , *et al*. (2019) Seawater salt‐trapped *Pseudomonas aeruginosa* survives for years and gets primed for salinity tolerance. BMC Microbiol 19: 142. 31234794 10.1186/s12866-019-1499-2 PMC6591848

[mbt213980-bib-0092] Elazari‐Volcani, B. (1940) Studies on the Microflora of the Dead Sea. PhD Thesis. The Hebrew University of Jerusalem, Hebrew, p. 5.

[mbt213980-bib-0093] Elliott, M.L. (2005) Survival, growth and pathogenicity of *Gaeumannomyces graminis* var. *graminis* with different methods of long‐term storage. Mycologia 97: 901–907. 16457359 10.3852/mycologia.97.4.901

[mbt213980-bib-0294] Estes, J. A. , Terborgh, J. , Brashares, J. S. , Power, M. E. , Berger, J. , Bond, W. J. , *et al.* (2011) Trophic downgrading of planet Earth. Science 333: 301–306. 21764740 10.1126/science.1205106

[mbt213980-bib-0094] Fallico, V. , Ross, R.P. , Fitzgerald, G.F. , and McAuliffe, O. (2011) Genetic response to bacteriophage infection in *Lactococcus lactis* reveals a four‐strand approach involving induction of membrane stress proteins, D‐alanylation of the cell wall, maintenance of proton motive force, and energy conservation. J Virol 85: 12032–12042. 21880765 10.1128/JVI.00275-11 PMC3209278

[mbt213980-bib-0095] Faria, M. , Hajek, A.E. , and Wraight, S.P. (2009) Imbibitional damage in conidia of the entomopathogenic fungi *Beauveria bassiana*, *Metarhizium acridum*, and *Metarhizium anisopliae* . Biol Control 51: 346–354.

[mbt213980-bib-0096] Fasuan, A.A. , Akin‐Obasola, B. , and Abiodun, B.O. (2021) Water activity relations of spoilage fungi associated with smoke‐dried catfish (*Clarias gariepinus*) sold in some open markets in Nigeria. J Food Sci Technol. 10.1007/s13197-021-05229-8. PMC9114268 35602448

[mbt213980-bib-0097] Fendrihan, S. , Dornmayr‐Pfaffenhuemer, M. , Gerbl, F.W. , Holzinger, A. , Grösbacher, M. , Briza, P. , *et al*. (2012) Spherical particles of halophilic archaea correlate with exposure to low water activity – implications for microbial survival in fluid inclusions of ancient halite. Geobiology 10: 424–433. 22804926 10.1111/j.1472-4669.2012.00337.x PMC3495301

[mbt213980-bib-0098] Feng, J. , Wang, C. , Lei, J. , Yang, Y. , Yan, Q. , Zhou, X. , *et al*. (2020) Warming‐induced permafrost thaw exacerbates tundra soil carbon decomposition mediated by microbial community. Microbiome 8: 3. 31952472 10.1186/s40168-019-0778-3 PMC6969446

[mbt213980-bib-0099] Foster, A.J. , Ryder, L.S. , Kershaw, M.J. , and Talbot, N.J. (2017) The role of glycerol in the pathogenic lifestyle of the rice blast fungus *Magnaporthe oryzae* . Environ Microbiol 19: 1008–1016. 28165657 10.1111/1462-2920.13688

[mbt213980-bib-0100] Foster, T. , Soreghan, G.S. , Soreghan, M.J. , Benison, K.C. , and Elmore, R.D. (2014) Climatic and palaeogeographic significance of aeolian sediment in the Middle Permian Dog Creek Shale (Midcontinent U.S.). Palaeogeogr Palaeoclimatol Palaeoecol 402: 12–29.

[mbt213980-bib-0101] García‐Corral, L.S. , Duarte, C.M. , and Agusti, S. (2020a) Impact of UV radiation on plankton net community production: responses in Western Australian estuarine and coastal waters. Mar Ecol Prog Ser 651: 45–56.

[mbt213980-bib-0102] García‐Corral, L.S. , Duarte, C.M. , and Agusti, S. (2020b) Plankton community metabolism in Western Australia: Estuarine, coastal and oceanic surface waters. Front Mar Sci 7: 1142.

[mbt213980-bib-0103] Garrett, A.J. , Casterline, M. & Salvaggio, C. (2010) Thermodynamics of partially frozen cooling lakes. Proc SPIE 7661, Thermosense XXXII. 766105. 10.1117/12.849349.

[mbt213980-bib-0104] Godin, P.J. , Stone, H. , Bahrami, R. , Schuerger, A.C. , and Moores, J.E. (2020) UV attenuation by Martian brines. Can J Phys 98: 567–570.

[mbt213980-bib-0105] Grabowski, N.T. , and Klein, G. (2017) Microbiology and foodborne pathogens in honey. Crit Rev Food Sci Nutr 57: 1852–1862. 26176586 10.1080/10408398.2015.1029041

[mbt213980-bib-0106] Grant, W.D. (2004) Life at low water activity. Philos Trans R Soc Lond B Biol Sci 359: 1249–1266. 15306380 10.1098/rstb.2004.1502 PMC1693405

[mbt213980-bib-0107] Grigorev, I.P. , and Korzhevskii, D.E. (2018) Current technologies for fixation of biological material for immunohistochemical analysis (review). Modern Technol Med 10: 156–165.

[mbt213980-bib-0108] Grimene, C. , Mghirbi, O. , Louvet, S. , Bord, J.P. , and Le Grusse, P. (2021) Spatial characterization of surface water vulnerability to diffuse pollution related to pesticide contamination: case of the Gimone watershed in France. Environ Sci Pollut Res Int. 10.1007/s11356-021-14253-2. 34036499

[mbt213980-bib-0109] Gunde‐Cimerman, N. , Plemenitaš, A. , and Oren, A. (2018) Strategies of adaptation of microorganisms of the three domains of life to high salt concentrations. FEMS Microbiol Rev 42: 353–375. 29529204 10.1093/femsre/fuy009

[mbt213980-bib-0110] Gura, C. , and Rogers, S.O. (2020) Metatranscriptomic and metagenomic analysis of biological diversity in subglacial Lake Vostok (Antarctica). Biology 9: 55. 10.3390/biology9030055 PMC7150893 32188079

[mbt213980-bib-0111] Hall, E.J. , and Giaccia, A.J. (2018) Radiobiology for the Radiologist, 8th edn. Philadelphia, PA: Lippincott Williams & Wilkins.

[mbt213980-bib-0112] Hallsworth, J.E. (2018) Stress‐free microbes lack vitality. Fungal Biol 122: 379–385. 29801781 10.1016/j.funbio.2018.04.003

[mbt213980-bib-0113] Hallsworth, J.E. (2019) Microbial unknowns at the saline limits for life. Nat Ecol Evol 3: 1503–1504. 31666739 10.1038/s41559-019-1021-0

[mbt213980-bib-0114] Hallsworth, J.E. (2021) Mars’ surface is not universally biocidal. Environ Microbiol 23: 3345–3350. 33780125 10.1111/1462-2920.15494

[mbt213980-bib-0115] Hallsworth, J.E. , Heim, S. , and Timmis, K.N. (2003) Chaotropic solutes cause water stress in *Pseudomonas putida* . Environ Microbiol 5: 1270–1280. 14641573 10.1111/j.1462-2920.2003.00478.x

[mbt213980-bib-0116] Hallsworth, J.E. , Koop, T. , Dallas, T.D. , Zorzano, M.‐P. , Burkhardt, J. , Golyshina, O.V. , *et al*. (2021b) Water activity in Venus’s uninhabitable clouds and other planetary atmospheres. Nat Astron 5: 665–675.

[mbt213980-bib-0117] Hallsworth, J.E. , and Magan, N. (1994) Improved biological control by changing polyols/trehalose in conidia of entomopathogens. In Brighton Crop Protection Council‐Pests and Diseases. British Crop Protection Council 1994. Farnham, UK, pp. 1091–1096.

[mbt213980-bib-0118] Hallsworth, J.E. , Mancinelli, R.L. , Conley, C.A. , Dallas, T.D. , Rinaldi, T. , Davila, A.F. , *et al*. (2021a) Astrobiology of life on Earth. Environ Microbiol 23: 3335–3344. 33817931 10.1111/1462-2920.15499

[mbt213980-bib-0119] Hallsworth, J.E. , Yakimov, M.M. , Golyshin, P.N. , Gillion, J.L.M. , D’Auria, G. , Alves, F.L. , *et al*. (2007) Limits of life in MgCl_2_‐containing environments: chaotropicity defines the window. Environ Microbiol 9: 803–813. 10.1111/j.1462-2920.2006.01212.x 17298378

[mbt213980-bib-0120] Harrison, J.P. , Dobinson, L. , Freeman, K. , McKenzie, R. , Wyllie, D. , Nixon, S.L. , and Cockell, C.S. (2015) Aerobically respiring prokaryotic strains exhibit a broader temperature–pH–salinity space for cell division than anaerobically respiring and fermentative strains. J R Soc Interface 12: 20150658. 26354829 10.1098/rsif.2015.0658 PMC4614477

[mbt213980-bib-0121] He, D. , Wu, F. , Ma, W. , Zhang, Y. , Gu, J.‐D. , Duan, Y. , *et al*. (2021) Insights into the bacterial and fungal communities and microbiome that causes a microbe outbreak on ancient wall paintings in the Maijishan Grottoes. Int Biodeterior 163: 105250.

[mbt213980-bib-0122] Henderson, L. (1913) The Fitness of the Environment. London, UK: Macmillan.

[mbt213980-bib-0123] Hibino, H. , Takai, M. , Noguchi, H. , Sawamura, S. , Takahashi, Y. , Saka, H. , and Shiku, H. (2017) An approach to the research on ion and water properties in the interphase between the plasma membrane and bulk extracellular solution. J Physiol Sci 67: 439–445. 28213824 10.1007/s12576-017-0530-3 PMC5594052

[mbt213980-bib-0124] Hoehler, T.M. , and Jørgensen, B.B. (2013) Microbial life under extreme energy limitation. Nat Rev Microbiol 11: 83–94. 23321532 10.1038/nrmicro2939

[mbt213980-bib-0125] Hooker, S.B. , Morrow, J.H. , and Matsuoka, A. (2013) Apparent optical properties of the Canadian Beaufort Sea – Part 2: The 1 % and 1 cm perspective in deriving and validating AOP data products. Biogeosciences 10: 4511–4527.

[mbt213980-bib-0126] Houskeeper, H.F. , Hooker, S.B. , and Kudela, R.M. (2021) Spectral range within global *a* _CDOM_(440) algorithms for oceanic, coastal, and inland waters with application to airborne measurements. Remote Sens Environ 253: 112155.

[mbt213980-bib-0127] Huby, T.J.C. , Clark, D.R. , McKew, B.A. , and McGenity, T.J. (2021) Extremely halophilic archaeal communities are resilient to short‐term entombment in halite. Environ Microbiol 23: 3370–3383. 31919959 10.1111/1462-2920.14913 PMC8359394

[mbt213980-bib-0128] Iacobellis, N.S. , and DeVay, J.E. (1986) Long term storage of plant‐pathogenic bacteria in sterile distilled water. Appl Environ Microbiol 52: 388–389. 16347141 10.1128/aem.52.2.388-389.1986 PMC203536

[mbt213980-bib-0129] Imachi, H. , Sakai, S. , Lipp, J.S. , Miyazaki, M. , Saito, Y. , Yamanaka, Y. , *et al*. (2014) *Pelolinea submarina* gen. nov., sp. nov., an anaerobic, filamentous bacterium of the phylum *Chloroflexi* isolated from subseafloor sediment. Int J Syst Evol Microbiol 64: 812–818. 24215824 10.1099/ijs.0.057547-0

[mbt213980-bib-0130] Jančič, S. , Zalar, P. , Kocev, D. , Schroers, H.J. , Džeroski, S. , and Gunde‐Cimerman, N. (2016) Halophily reloaded: new insights into the extremophilic life‐style of *Wallemia* with the description of *Wallemia hederae* sp. nov. Fungal Divers 76: 97–118.

[mbt213980-bib-0131] Javor, B.J. (1984) Growth potential of halophilic bacteria isolated from solar salt environments: carbon sources and salt requirements. Appl Environ Microbiol 48: 352–360. 16346609 10.1128/aem.48.2.352-360.1984 PMC241517

[mbt213980-bib-0132] Jia, Z. , Wang, Z. , and Wang, H. (2019) Characteristics of dew formation in the semi‐arid Loess Plateau of central Shaanxi Province, China. Water 11: 126.

[mbt213980-bib-0133] Johnson, S.S. , Chevrette, M.G. , Ehlmann, B.L. , and Benison, K.C. (2015) Insights from the metagenome of an acid salt lake: the role of biology in an extreme depositional environment. PLoS One 10: e0122869. 25923206 10.1371/journal.pone.0122869 PMC4414474

[mbt213980-bib-0134] Johnson, S.S. , Millan, M. , Graham, H. , Benison, K.C. , Williams, A.J. , McAdam, A. , *et al*. (2020) Lipid biomarkers in ephemeral acid salt lake mudflat/sandflat sediments: implications for Mars. Astrobiology 20: 167–178. 32022603 10.1089/ast.2017.1812

[mbt213980-bib-0135] Jørgensen, B.B. , and Marshall, I.P.G. (2016) Slow microbial life in the seabed. Annu Rev Mar Sci 8: 311–332. 10.1146/annurev-marine-010814-015535 26209150

[mbt213980-bib-0136] Kanô, F. , Hashimoto, M. , Kaminoh, Y. , and Ueda, I. (1994) Theory for phase transition of phospholipid multilayer systems at low water content. Jpn J Appl Phys 33: 5109–5117.

[mbt213980-bib-0137] Karan, R. , Mathew, S. , Muhammad, R. , Bautista, D.B. , Vogler, M. , Eppinger, J. , *et al*. (2020) Understanding high‐salt and cold adaptation of a polyextremophilic enzyme. Microorganisms 8: 1594. 10.3390/microorganisms8101594 PMC7602713 33081237

[mbt213980-bib-0138] Kates, M. (1993) Biology of halophilic bacteria, Part II. Membrane lipids of extreme halophiles: biosynthesis, function and evolutionary significance. Experientia 49: 1027–1036. 8270029 10.1007/BF01929909

[mbt213980-bib-0139] Kelly, T.B. , Knapp, A.N. , Landry, M.R. , Selph, K.E. , Shropshire, T.A. , Thomas, R.K. , and Stukel, M.R. (2021) Lateral advection supports nitrogen export in the oligotrophic open‐ocean Gulf of Mexico. Nat Commun 12: 3325. 34083545 10.1038/s41467-021-23678-9 PMC8175579

[mbt213980-bib-0140] Khlaifat, A. , Hogan, M. , Phillips, G. , Nawayseh, K. , Amira, J. , and Talafeha, E. (2010) Long‐term monitoring of the Dead Sea level and brine physico‐chemical parameters “from 1987 to 2008”. J Mar Syst 81: 207–212.

[mbt213980-bib-0141] Kirdyanov, A.V. , Saurer, M. , Siegwolf, R. , Knorre, A.A. , Prokushkin, A.S. , Churakova (Sidorova), O.V. , *et al*. (2020) Long‐term ecological consequences of forest fires in the continuous permafrost zone of Siberia. Environ Res Lett 15: 034061.

[mbt213980-bib-0142] Kluge, M. , Wauthy, M. , Clemmensen, K.E. , Wurzbacher, C. , Hawkes, J.A. , Einarsdottir, K. , *et al*. (2021) Declining fungal diversity in Arctic freshwaters along a permafrost thaw gradient. Glob Chang Biol. 27: 5889–5906. 10.1111/gcb.15852. 34462999

[mbt213980-bib-0143] Kochkina, G. , Ivanushkina, N. , Ozerskaya, S. , Chigineva, N. , Vasilenko, O. , Firsov, S. , *et al*. (2012) Ancient fungi in Antarctic permafrost environments. FEMS Microbiol Ecol 82: 501–509. 22757669 10.1111/j.1574-6941.2012.01442.x

[mbt213980-bib-0144] Kreuzer‐Martin, H.W. , Ehrlinger, J.R. , and Hegg, E.L. (2005) Oxygen isotopes indicate most intracellular water in log‐phase *Escherichia coli* is derived from metabolism. Proc Natl Acad Sci USA 102: 17337–17341. 16301541 10.1073/pnas.0506531102 PMC1297667

[mbt213980-bib-0145] Kreuzer‐Martin, H.W. , Lott, M.J. , Ehleringer, J.R. , and Hegg, E.L. (2006) Metabolic processes account for the majority of the intracellular water in log‐phase *Escherichia coli* cells as revealed by hydrogen isotopes. Biochemistry 45: 13622–13630. 17087516 10.1021/bi0609164

[mbt213980-bib-0146] Krisko, A. , and Radman, M. (2010) Protein damage and death by radiation in *Escherichia coli* and *Deinococcus radiodurans* . Proc Natl Acad Sci USA 107: 14373–14377. 20660760 10.1073/pnas.1009312107 PMC2922536

[mbt213980-bib-0147] Kruuv, J. , Lepock, J.R. , and Keith, A.D. (1978) The effect of fluidity of membrane lipids on freeze‐thaw survival of yeast. Cryobiology 15: 73–79. 342200 10.1016/0011-2240(78)90009-3

[mbt213980-bib-0148] La Cono, V. , Bortoluzzi, G. , Messina, E. , La Spada, G. , Smedile, F. , Giuliano, L. , *et al*. (2019) The discovery of Lake *Hephaestus*, the youngest athalassohaline deep‐sea formation on Earth. Sci Rep 9: 1679. 30737448 10.1038/s41598-018-38444-z PMC6368551

[mbt213980-bib-0149] LaRowe, D.E. , and Amend, J.P. (2015) Power limits for microbial life. Front Microbiol 6: 718. 26236299 10.3389/fmicb.2015.00718 PMC4502533

[mbt213980-bib-0150] Lebre, P.H. , De Maayer, P. , and Cowan, D.A. (2017) Xerotolerant bacteria: surviving through a dry spell. Nat Rev Microbiol 15: 285–296. 28316329 10.1038/nrmicro.2017.16

[mbt213980-bib-0151] Lee, C.J.D. , McMullan, P.E. , O’Kane, C.J. , Stevenson, A. , Santos, I.C. , Roy, C. , *et al*. (2018) NaCl‐saturated brines are thermodynamically moderate, rather than extreme, microbial habitats. FEMS Microbiol Rev 42: 672–693. 29893835 10.1093/femsre/fuy026

[mbt213980-bib-0152] Li, H. , Yu, C. , Wang, F. , Chang, S.J. , Yao, J. , and Blake, R.E. (2016) Probing the metabolic water contribution to intracellular water using oxygen isotope ratios of PO_4_ . Proc Natl Acad Sci USA 113: 5862–5867. 27170190 10.1073/pnas.1521038113 PMC4889363

[mbt213980-bib-0153] Liang, R. , Lau, M. , Vishnivetskaya, T. , Lloyd, K.G. , Wang, W. , Wiggins, J. , *et al*. (2019) Predominance of anaerobic, spore‐forming bacteria in metabolically active microbial communities from ancient Siberian permafrost. Appl Environ Microbiol 85: e00560‐19. 31152014 10.1128/AEM.00560-19 PMC6643238

[mbt213980-bib-0154] Liang, R. , Li, Z. , Lau Vetter, M.C.Y. , Vishnivetskaya, T.A. , Zanina, O.G. , Lloyd, K.G. , *et al*. (2021) Genomic reconstruction of fossil and living microorganisms in ancient Siberian permafrost. Microbiome 9: 110. 34001281 10.1186/s40168-021-01057-2 PMC8130349

[mbt213980-bib-0155] Liao, C.H. , and Shollenberger, L.M. (2003) Survivability and long‐term preservation of bacteria in water and in phosphate‐buffered saline. Lett Appl Microbiol 37: 45–50. 12803555 10.1046/j.1472-765x.2003.01345.x

[mbt213980-bib-0156] Lievens, B. , Hallsworth, J. E. , Pozo, M. I. , Belgacem, Z. B. , Stevenson, A. , Willems, K. A. , and Jacquemyn, H. (2015) Microbiology of sugar‐rich environments: diversity, ecology and system constraints. Environ Microbiol 17: 278–298. 25041632 10.1111/1462-2920.12570

[mbt213980-bib-0157] Lomsadze, G. (2012) Report: Georgia unearths the world’s oldest honey [WWW document]. URL http://www.eurasianet.org/node/65204.

[mbt213980-bib-0158] Lomstein, B.A. , Langerhuus, A.T. , D’Hondt, S. , Jørgensen, B.B. , and Spivack, A. (2012) Endospore abundance, microbial growth and necromass turnover in deep sub‐seafloor sediment. Nature 484: 101–104. 22425999 10.1038/nature10905

[mbt213980-bib-0159] Maccario, L. , Sanguino, L. , Vogel, T.M. , and Larose, C. (2015) Snow and ice ecosystems: not so extreme. Res Microbiol 166: 782–795. 26408452 10.1016/j.resmic.2015.09.002

[mbt213980-bib-0160] Marsh, N.B. , Lacelle, D. , Faucher, B. , Cotroneo, S. , Jasperse, L. , Clark, I.D. , and Andersen, D.T. (2020) Sources of solutes and carbon cycling in perennially ice‐covered Lake Untersee, Antarctica. Sci Rep 10: 12290. 32704043 10.1038/s41598-020-69116-6 PMC7378197

[mbt213980-bib-0161] Maughan, H. , Birky, C.W. Jr , Nicholson, W.L. , Rosenzweig, W.D. , and Vreeland, R.H. (2002) The paradox of the “ancient” bacterium which contains “modern” protein‐coding genes. Mol Biol Evol 19: 1637–1639. 12200492 10.1093/oxfordjournals.molbev.a004227

[mbt213980-bib-0162] Maurer, M. , and Oostenbrink, C. (2019) Water in protein hydration and ligand recognition. J Mol Recognit 32: e2810. 31456282 10.1002/jmr.2810 PMC6899928

[mbt213980-bib-0163] McCammick, E.M. , Gomase, V.S. , Timson, D.J. , McGenity, T.J. , and Hallsworth, J.E. (2010) Water‐hydrophobic compound interactions with the microbial cell. In Handbook of Hydrocarbon and Lipid Microbiology – Hydrocarbons, Oils and Lipids: Diversity, Properties and Formation, vol 2, Timmis, K.N. (ed). New York, NY, USA: Springer, pp. 1451–1466.

[mbt213980-bib-0164] McGenity, T.J. , Gemmell, R.T. , Grant, W.D. , and Stan‐Lotter, H. (2000) Origins of halophilic microorganisms in ancient salt deposits. Environ Microbiol 2: 243–250. 11200425 10.1046/j.1462-2920.2000.00105.x

[mbt213980-bib-0165] Meisner, A. , Snoek, B.L. , Nesme, J. , Dent, E. , Jacquiod, S. , Classen, A.T. , and Priemé, A. (2021) Soil microbial legacies differ following drying‐rewetting and freezing‐thawing cycles. ISME J 15: 1207–1221. 33408369 10.1038/s41396-020-00844-3 PMC8115648

[mbt213980-bib-0166] Mhatre, S.S. , Kaufmann, S. , Marshall, I.P.G. , Obrochta, S. , Andrén, T. , Jørgensen, B.B. , and Lomstein, B.A. (2019) Microbial biomass turnover times and clues to cellular protein repair in energy‐limited deep Baltic Sea sediments. FEMS Microbiol Ecol 95: fiz068. 31095297 10.1093/femsec/fiz068

[mbt213980-bib-0167] Mikhailov, E. , Vlasenko, S. , Niessner, R. , and Pöschl, U. (2004) Interaction of aerosol particles composed of protein and salts with water vapor: hygroscopic growth and microstructural rearrangement. Atmos Chem Phys 4: 323–350.

[mbt213980-bib-0168] Miller, S.L. , and Urey, H.C. (1959) Organic compound synthesis on the primitive Earth. Science 130: 245–251. 13668555 10.1126/science.130.3370.245

[mbt213980-bib-0169] Mißbach, H. , Duda, J.P. , van den Kerkhof, A.M. , Lüders, V. , Pack, A. , Reitner, J. , and Thiel, V. (2021) Ingredients for microbial life preserved in 3.5 billion‐year‐old fluid inclusions. Nat Commun 12: 1101. 33597520 10.1038/s41467-021-21323-z PMC7889642

[mbt213980-bib-0170] Miteva, V.I. , Sheridan, P.P. , and Brenchley, J.E. (2004) Phylogenetic and physiological diversity of microorganisms isolated from a deep Greenland glacier ice core. Appl Environ Microbiol 70: 202–213. 14711643 10.1128/AEM.70.1.202-213.2004 PMC321287

[mbt213980-bib-0171] Moger‐Reischer, R.Z. , and Lennon, J.T. (2019) Microbial ageing and longevity. Nat Rev Microbiol 17: 679–690. 31534207 10.1038/s41579-019-0253-y

[mbt213980-bib-0172] Morley, C.R. , Trofymow, J.A. , Coleman, D.C. , and Cambardella, C. (1983) Effects of freeze‐thaw stress on bacterial populations in soil microcosms. Microb Ecol 9: 329–340. 24221821 10.1007/BF02019022

[mbt213980-bib-0173] Mormile, M.R. , Biesen, M.A. , Gutierrez, M.C. , Ventosa, A. , Pavlovich, J.B. , Onstott, T.C. , and Fredrickson, J.K. (2003) Isolation of *Halobacterium salinarum* retrieved directly from halite brine inclusions. Environ Microbiol 5: 1094–1102. 14641589 10.1046/j.1462-2920.2003.00509.x

[mbt213980-bib-0174] Mormile, M.R. , Hong, B.Y. , and Benison, K.C. (2009) Molecular analysis of the microbial communities of Mars analog lakes in Western Australia. Astrobiology 9: 919–930. 20041745 10.1089/ast.2008.0293

[mbt213980-bib-0175] Morono, Y. , Ito, M. , Hoshino, T. , Terada, T. , Hori, T. , Ikehara, M. , *et al*. (2020) Aerobic microbial life persists in oxic marine sediment as old as 101.5 million years. Nat Commun 11: 3626. 32724059 10.1038/s41467-020-17330-1 PMC7387439

[mbt213980-bib-0176] Morono, Y. , Terada, T. , Nishizawa, M. , Ito, M. , Hillion, F. , Takahata, N. , *et al*. (2011) Carbon and nitrogen assimilation in deep subseafloor microbial cells. Proc Natl Acad Sci USA 108: 18295–18300. 21987801 10.1073/pnas.1107763108 PMC3215001

[mbt213980-bib-0177] Mou, Y.Z. , Qiu, X.X. , Zhao, M.L. , Cui, H.L. , Oh, D. , and Dyall‐Smith, M.D. (2012) *Halohasta litorea* gen. nov. sp. nov., and *Halohasta litchfieldiae* sp. nov., isolated from the Daliang aquaculture farm, China and from Deep Lake, Antarctica, respectively. Extremophiles 16: 895–901. 23052830 10.1007/s00792-012-0485-5

[mbt213980-bib-0178] Muldrew, K. , and McGann, L.E. (1990) Mechanisms of intracellular ice formation. Biophys J 57: 525–532. 2306499 10.1016/S0006-3495(90)82568-6 PMC1280746

[mbt213980-bib-0179] Muldrew, K. , and McGann, L.E. (1994) The osmotic rupture hypothesis of intracellular freezing injury. Biophys J 66: 532–541. 8161706 10.1016/s0006-3495(94)80806-9 PMC1275720

[mbt213980-bib-0180] Mulyukin, A.L. , Demkina, E.V. , Manucharova, N.A. , Akimov, V.N. , Andersen, D. , McKay, C. , and Gal'chenko, V.F. (2014) Prokaryotic community of subglacial bottom sediments of Antarctic Lake Untersee: Detection by cultural and direct microscopic techniques. Microbiology 83: 77–84. 25423725

[mbt213980-bib-0181] Murthy, M.R.N. (2021) Protein hydration. Curr Sci 120: 186–192.

[mbt213980-bib-0182] Najjari, A. , Stathopoulou, P. , Elmnasri, K. , Hasnaoui, F. , Zidi, I. , Sghaier, H. , *et al*. (2021) Assessment of 16S rRNA gene‐based phylogenetic diversity of Archaeal communities in halite‐crystal salts processed from natural Saharan saline systems of Southern Tunisia. Biology 10: 397. 34064384 10.3390/biology10050397 PMC8147861

[mbt213980-bib-0183] Nelson, J.A. , Pérez‐Priego, O. , Zhou, S. , Poyatos, R. , Zhang, Y. , Blanken, P.D. , *et al*. (2020) Ecosystem transpiration and evaporation: Insights from three water flux partitioning methods across FLUXNET sites. Glob Change Biol 26: 6916–6930. 10.1111/gcb.15314 33022860

[mbt213980-bib-0184] Ng, F. , Hallet, B. , Sletten, R.S. , and Stone, J.O. (2005) Fast‐growing till over ancient ice in Beacon Valley, Antarctica. Geology 33: 121–124.

[mbt213980-bib-0185] Ng, T.W. , Chan, W.L. , and Lai, K.M. (2017) Importance of stress‐response genes to the survival of airborne *Escherichia coli* under different levels of relative humidity. AMB Exp 7: 71. 10.1186/s13568-017-0376-3 PMC5366994 28342170

[mbt213980-bib-0186] Nikitaki, Z. , Mavragani, I.V. , Laskaratou, D.A. , Gika, V. , Moskvin, V.P. , Theofilatos, K. , *et al*. (2016) Systemic mechanisms and effects of ionizing radiation: A new 'old' paradigm of how the bystanders and distant can become the players. Semin Cancer Biol 37–38: 77–95. 10.1016/j.semcancer.2016.02.002 26873647

[mbt213980-bib-0187] Nomura, Y. , Ito, S. , Teranishi, M. , Ono, H. , Inoue, K. , and Kandori, H. (2018) Low‐temperature FTIR spectroscopy provides evidence for protein‐bound water molecules in eubacterial light‐driven ion pumps. Phys Chem Chem Phys 20: 3165–3171. 28975940 10.1039/c7cp05674e

[mbt213980-bib-0188] Ogilvie, J. (1868) The Imperial Dictionary; English, Technological, and Scientific, on the Basis of Webster’s English Dictionary with the Addition of Many Thousand Words and Phrases Including the Most Generally Used Technical and Scientific Terms with Their Etymology and Their Pronunciation. London, UK: Blackie and Son.

[mbt213980-bib-0189] Olson, D.G. , Sparling, R. , and Lynd, L.R. (2015) Ethanol production by engineered thermophiles. Curr Opin Biotechnol 33: 130–141. 25745810 10.1016/j.copbio.2015.02.006

[mbt213980-bib-0190] Oren, A. (1998) Life and survival in a magnesium chloride brine: the biology of the Dead Sea. Proc SPIE 3441 Instrum Meth Missions Astrobiol 44–54.

[mbt213980-bib-0191] Orsi, W.D. , Edgcomb, V.P. , Christman, G.D. , and Biddle, J.F. (2013) Gene expression in the deep biosphere. Nature 499: 205–208. 23760485 10.1038/nature12230

[mbt213980-bib-0192] Overmans, S. , and Agustí, S. (2019) Latitudinal gradient of UV attenuation along the highly transparent Red Sea basin. Photochem Photobiol 95: 1267–1279. 31066904 10.1111/php.13112 PMC6852308

[mbt213980-bib-0193] Pachler, M. , Kabelka, I. , Appavou, M.S. , Lohner, K. , Vácha, R. , and Pabst, G. (2019) Magainin 2 and PGLa in bacterial membrane mimics I: Peptide‐peptide and lipid‐peptide interactions. Biophys J 117: 1858–1869. 31703802 10.1016/j.bpj.2019.10.022 PMC7031808

[mbt213980-bib-0194] Padmavathy, V. , Vasudevan, P. , and Dhingra, S.C. (2003) Thermal and spectroscopic studies on sorption of nickel(II) ion on protonated baker's yeast. Chemosphere 52: 1807–1817. 12871747 10.1016/S0045-6535(03)00222-4

[mbt213980-bib-0195] Panikova, N.S. , Flanagan, P.W. , Oechel, W.C. , Mastepanov, M.A. , and Christensen, T.R. (2006) Microbial activity in soils frozen to below ‐39°C. Soil Biol Biochem 38: 785–794.

[mbt213980-bib-0196] Park, J.I. , Grant, C.M. , Attfield, P.V. , and Dawes, I.W. (1997) The freeze‐thaw stress response of the yeast *Saccharomyces cerevisiae* is growth phase specific and is controlled by nutritional state via the *RAS*‐cyclic AMP signal transduction pathway. Appl Environ Microbiol 63: 3818–3824. 9327544 10.1128/aem.63.10.3818-3824.1997 PMC168690

[mbt213980-bib-0197] Patel, A.R. , and Frank, C.W. (2006) Quantitative analysis of tethered vesicle assemblies by quartz crystal microbalance with dissipation monitoring: Binding dynamics and bound water content. Langmuir 22: 7587–7599. 16922537 10.1021/la0610452

[mbt213980-bib-0198] Pavankumar, T.L. , Mittal, P. , and Hallsworth, J.E. (2021) Molecular insights into the ecology of a psychrotolerant *Pseudomonas syringae* . Environ Microbiol 23: 3665–3681. 33169927 10.1111/1462-2920.15304

[mbt213980-bib-0199] Pedrós‐Alió, C. (2021) Time travel in microorganisms. Syst Appl Microbiol 44: 126227. 34252729 10.1016/j.syapm.2021.126227

[mbt213980-bib-0200] Pérez‐Ruiz, E.R. , Vivoni, E.R. , Yépez, E.A. , Rodríguez, J.C. , Gochis, D.J. , Robles‐Morua, A. , *et al*. (2021) Landscape controls on water‐energy‐carbon fluxes across different ecosystems during the North American monsoon. J Geophys Res Biogeosci 126: e2020JG005809.

[mbt213980-bib-0201] Pikal‐Cleland, K.A. , and Carpenter, J.F. (2001) Lyophilization‐induced protein denaturation in phosphate buffer systems: monomeric and tetrameric β‐galactosidase. J Pharm Sci 90: 1255–1268. 11745778 10.1002/jps.1078

[mbt213980-bib-0202] Potts, M. (1994) Desiccation tolerance of prokaryotes. Microbiol Rev 58: 755–805. 7854254 10.1128/mr.58.4.755-805.1994 PMC372989

[mbt213980-bib-0203] Price, P.B. (2000) A habitat for psychrophiles in deep Antarctic ice. Proc Natl Acad Sci USA 97: 1247–1251. 10655516 10.1073/pnas.97.3.1247 PMC15584

[mbt213980-bib-0204] Price, P.B. (2009) Microbial genesis, life and death in glacial ice. Can J Microbiol 55: 1–11. 19190696 10.1139/W08-117

[mbt213980-bib-0205] Price, P.B. , and Sowers, T. (2004) Temperature dependence of metabolic rates for microbial growth, maintenance, and survival. Proc Natl Acad Sci USA 101: 4631–4636. 15070769 10.1073/pnas.0400522101 PMC384798

[mbt213980-bib-0206] Qiu, L. , Zhang, M. , Tang, J. , Adhikari, B. , and Cao, P. (2019) Innovative technologies for producing and preserving intermediate moisture foods: a review. Food Res Int 116: 90–102. 30717022 10.1016/j.foodres.2018.12.055

[mbt213980-bib-0207] Radman, M. (2016) Protein damage, radiation sensitivity and aging. DNA Repair 44: 186–192. 27264559 10.1016/j.dnarep.2016.05.025

[mbt213980-bib-0308] Ramos‐Barbero, M. D. , Viver, T. , Zabaleta, A. , Senel, E. , and Gomariz, M. et al. (2021) Ancient saltern metagenomics: tracking changes in microbes and their viruses from the underground to the surface. Environ Microbiol 23: 3477–3498. 34110059 10.1111/1462-2920.15630

[mbt213980-bib-0208] Ranawat, P. , and Rawat, S. (2017) Radiation resistance in thermophiles: mechanisms and applications. World J Microbiol Biotechnol 33: 112. 28470425 10.1007/s11274-017-2279-5

[mbt213980-bib-0209] Rapoport, A. (2019) Anhydrobiosis in non‐conventional yeasts. In Non‐Conventional Yeasts: From Basic Research to Application. Sibirny, A. (ed.). Berlin/Heidelberg, Germany: Springer International Publishing, pp. 341–359.

[mbt213980-bib-0210] Rapoport, A. , Golovina, E.A. , Gervais, P. , Dupont, S. , and Beney, L. (2019) Anhydrobiosis: inside yeast cells. Biotechnol Adv 37: 51–67. 30453013 10.1016/j.biotechadv.2018.11.003

[mbt213980-bib-0211] Rapoport, A.I. , Khroustalyova, G.M. , Camanis, G.J. , and Beker, M.J. (1995) Yeast anhydrobiosis: permeability of the plasma membrane. Microbiology 64: 229–232. 7616882

[mbt213980-bib-0212] Rapp, J.Z. , Sullivan, M.B. , and Deming, J.W. (2021) Divergent genomic adaptations in the microbiomes of Arctic subzero sea‐ice and cryopeg brines. Front Microbiol 12: 701186. 34367102 10.3389/fmicb.2021.701186 PMC8339730

[mbt213980-bib-0213] Rech, S. , Gusmao, J.B. , Kiessling, T. , Hidalgo‐Ruz, V. , Meerhoff, E. , Gatta‐Rosemary, M. , *et al*. (2021) A desert in the ocean ‐ Depauperate fouling communities on marine litter in the hyper‐oligotrophic South Pacific Subtropical Gyre. Sci Total Environ 759: 143545. 33203559 10.1016/j.scitotenv.2020.143545

[mbt213980-bib-0214] Revich, B. , Tokarevich, N. , and Parkinson, A.J. (2012) Climate change and zoonotic infections in the Russian Arctic. Internat J Circumpolar Health 71: 18792. 10.3402/ijch.v71i0.18792 PMC3417549 22868189

[mbt213980-bib-0215] Rhoads, W.J. , Ji, P. , Pruden, A. , and Edwards, M.A. (2015) Water heater temperature set point and water use patterns influence *Legionella pneumophila* and associated microorganisms at the tap. Microbiome 3: 67. 26627188 10.1186/s40168-015-0134-1 PMC4666224

[mbt213980-bib-0216] Rivkina, E. , Gilichinsky, D. , Wagener, S. , Tiedje, J. , and McGrath, J. (1998) Biogeochemical activity of anaerobic microorganisms from buried permafrost sediments. Geomicrobiol J 15: 187–193.

[mbt213980-bib-0217] Roark, M. , and Feller, S.E. (2008) Structure and dynamics of a fluid phase bilayer on a solid support as observed by a molecular dynamics computer simulation. Langmuir 24: 12469–12473. 18850686 10.1021/la802079h PMC2632950

[mbt213980-bib-0218] Robock, A. (2000) Volcanic eruptions and climate. Rev Geophys 38: 191–219.

[mbt213980-bib-0219] Rose, K.C. , Neale, P.J. , Tzortziou, M. , Gallegos, C.L. , and Jordan, T.E. (2019) Patterns of spectral, spatial, and long‐term variability in light attenuation in an optically complex sub‐estuary. Limnol Oceanogr 64: S257–S272.

[mbt213980-bib-0220] Rosinger, C. , and Bonkowski, M. (2021) Soil age and soil organic carbon content shape biochemical responses to multiple freeze–thaw events in soils along a postmining agricultural chronosequence. Biogeochemistry 155: 113–125.

[mbt213980-bib-0080] Rubin, S.S. , Marín, I. , Gómez, M.J. , Morales, E.A. , Zekker, I. , San Martín‐Uriz, P. , *et al*. (2017) Prokaryotic diversity and community composition in the Salar de Uyuni, a large scale, chaotropic salt flat. Environ Microbiol 19: 3745–3754. 28752915 10.1111/1462-2920.13876

[mbt213980-bib-0221] Rummel, J.D. , Beaty, D.W. , Jones, M.A. , Bakermans, C. , Barlow, N.G. , Boston, P.J. , *et al*. (2014) A new analysis of Mars “special regions”: findings of the second MEPAG Special Regions Science Analysis Group (SR‐SAG2). Astrobiology 14: 887–968. 25401393 10.1089/ast.2014.1227

[mbt213980-bib-0222] Sahoo, R.R. , Ghosh, P. , and Sarkar, J. (2017) Energy and exergy comparisons of water based optimum brines as coolants for rectangular fin automotive radiator. Int J Heat Mass Transf 105: 690–696.

[mbt213980-bib-0223] Sano, F. , Asakawa, N. , Inoue, Y. , and Sakurai, M. (1999) A dual role for intracellular trehalose in the resistance of yeast cells to water stress. Cryobiology 39: 80–87. 10458903 10.1006/cryo.1999.2188

[mbt213980-bib-0224] Santos, R. , Stevenson, A. , de Carvalho, C.C.C.R. , Grant, I.R. , and Hallsworth, J.E. (2015) Extraordinary solute stress tolerance contributes to the environmental tenacity of mycobacteria. Environ Microbiol Rep 7: 746–764. 26059202 10.1111/1758-2229.12306

[mbt213980-bib-0225] Sanz, J.L. , Rodríguez, N. , Escudero, C. , Carrizo, D. , Amils, R. , and Gómez, F. (2021) Methanogenesis at high temperature, high ionic strength and low pH in the volcanic Aaea of Dallol, Ethiopia. Microorganisms 9: 1231. 34204110 10.3390/microorganisms9061231 PMC8228321

[mbt213980-bib-0226] Satheesh, S. , and Krishnamoorthy, K. (2005) Radiative effects of natural aerosols: a review. Atmos Environ 39: 2089–2110.

[mbt213980-bib-0227] Schimel, J. , Balser, T.C. , and Wallenstein, M. (2007) Microbial stress‐response physiology and its implications for ecosystem function. Ecology 88: 1386–1394. 17601131 10.1890/06-0219

[mbt213980-bib-0228] Schreder‐Gomes, S.I. & Benison, K.C. (2021) Optical recognition of 830 million year old microorganisms trapped in bedded halite: Implications for future return samples from Mars. LPI Contrib. No. 2548. PDF 1072. 52nd Lunar and Planetary Science Conference 2021, The Woodlands, Texas.

[mbt213980-bib-0229] Schreder‐Gomes, S. , Benison, K.C. , and Bernau, J. (2021) 830‐million‐year‐old microorganisms in primary fluid inclusions in halite. Geology In press.

[mbt213980-bib-0230] Schreiber, U. , Mayer, C. , Schmitz, O.J. , Rosendahl, P. , Bronja, A. , Greule, M. , *et al*. (2017) Organic compounds in fluid inclusions of Archean quartz—Analogues of prebiotic chemistry on early Earth. PLoS One 12: e0177570. 28614348 10.1371/journal.pone.0177570 PMC5470662

[mbt213980-bib-0231] Schubert, B.A. , Lowenstein, T.K. , Timofeeff, M.N. , and Parker, M.A. (2010) Halophilic *Archaea* cultured from ancient halite, Death Valley, California. Environ Microbiol 12: 440–454. 19840101 10.1111/j.1462-2920.2009.02086.x

[mbt213980-bib-0232] Shoemaker, W.R. , Jones, S.E. , Muscarella, M.E. , Behringer, M.G. , Lehmkuhl, B.K. , and Lennon, J.T. (2021) Microbial population dynamics and evolutionary outcomes under extreme energy limitation. Proc Natl Acad Sci USA 118: e2101691118. 34385301 10.1073/pnas.2101691118 PMC8379937

[mbt213980-bib-0233] Siegert, M. , Ellis‐Evans, J. , Tranter, M. , Mayer, C. , Petit, J.R. , Salamatin, A. , and Priscu, J.C. (2001) Physical, chemical and biological processes in Lake Vostok and other Antarctic subglacial lakes. Nature 414: 603–609. 11740551 10.1038/414603a

[mbt213980-bib-0234] Sipes, K. , Almatari, A. , Eddie, A. , Williams, D. , Spirina, E. , Rivkina, E. , *et al*. (2021) Eight metagenome‐assembled genomes provide evidence for microbial adaptation in 20,000‐ to 1,000,000‐year‐old Siberian permafrost. Appl Environ Microbiol 87: e0097221. 34288700 10.1128/AEM.00972-21 PMC8432575

[mbt213980-bib-0235] Slieman, T.A. , and Nicholson, W.L. (2000) Artificial and solar UV radiation induces strand breaks and cyclobutane pyrimidine dimers in *Bacillus subtilis* spore DNA. Appl Environ Microbiol 66: 199–205. 10618224 10.1128/aem.66.1.199-205.2000 PMC91806

[mbt213980-bib-0236] Smith, M.D. , and Smith, J.C. (2020) Effects of sodium and calcium chloride ionic stresses on model yeast membranes revealed by molecular dynamics simulation. Chem Phys Lipids 233: 104980. 33038307 10.1016/j.chemphyslip.2020.104980

[mbt213980-bib-0237] Steinle, L. , Knittel, K. , Felber, N. , Casalino, C. , de Lange, G. , Tessarolo, C. , *et al*. (2018) Life on the edge: active microbial communities in the Kryos MgCl_2_‐brine basin at very low water activity. ISME J 12: 1414–1426. 29666446 10.1038/s41396-018-0107-z PMC5956074

[mbt213980-bib-0238] Stevenson, A. , Burkhardt, J. , Cockell, C.S. , Cray, J.A. , Dijksterhuis, J. , Fox‐Powell, M. , *et al*. (2015b) Multiplication of microbes below 0.690 water activity: implications for terrestrial and extraterrestrial life. Environ Microbiol 2: 257–277. 10.1111/1462-2920.12598 25142751

[mbt213980-bib-0239] Stevenson, A. , Cray, J.A. , Williams, J.P. , Santos, R. , Sahay, R. , Neuenkirchen, N. , *et al*. (2015a) Is there a common water‐activity limit for the three domains of life? ISME J 9: 1333–1351. 25500507 10.1038/ismej.2014.219 PMC4438321

[mbt213980-bib-0240] Stevenson, A. , Hamill, P.G. , O’Kane, C.J. , Kminek, G. , Rummel, J.D. , Voytek, M.A. , *et al*. (2017) *Aspergillus penicillioides* differentiation and cell division at 0.585 water activity. Environ Microbiol 19: 687–697. 27871132 10.1111/1462-2920.13597

[mbt213980-bib-0241] Sugden, D.E. , Marchant, D.R. , Potter, N. , Souchez, R.A. , Denton, G.H. , Swisher, C.C. III , and Tison, J.‐L. (1995) Preservation of Miocene glacier ice in East Antarctica. Nature 376: 412–414.

[mbt213980-bib-0242] Suzuki, Y. , Yamashita, S. , Kouduka, M. , Ao, Y. , Mukai, H. , Mitsunobu, S. , *et al*. (2020) Deep microbial proliferation at the basalt interface in 33.5‐104 million‐year‐old oceanic crust. Commun Biol 3: 136. 32242062 10.1038/s42003-020-0860-1 PMC7118141

[mbt213980-bib-0243] Takamura, K. , Fischer, H. , and Morrow, N.R. (2012) Physical properties of aqueous glycerol solutions. J Pet Sci Eng 98–99: 50–60.

[mbt213980-bib-0244] Tang, J.W. (2009) The effect of environmental parameters on the survival of airborne infectious agents. J R Soc Interface 6: S737–S746. 19773291 10.1098/rsif.2009.0227.focus PMC2843949

[mbt213980-bib-0245] Tanghe, A. , Van Dijck, P. , Colavizza, D. , and Thevelein, J.M. (2004) Aquaporin‐mediated improvement of freeze tolerance of *Saccharomyces cerevisiae* is restricted to rapid freezing conditions. Appl Environ Microbiol 70: 3377–3382. 15184134 10.1128/AEM.70.6.3377-3382.2004 PMC427737

[mbt213980-bib-0246] Tedetti, M. , and Sempéré, R. (2006) Penetration of ultraviolet radiation in the marine environment: a review. Photochem Photobiol 82: 389–397. 16613490 10.1562/2005-11-09-IR-733

[mbt213980-bib-0247] Tereshina, V.M. , Memorskaia, A.S. , Kotlova, E.R. , and Feofilova, E.P. (2010) Membrane lipid and cytosol carbohydrate composition in *Aspergillus niger* under heat shock. Microbiology 79: 40–46. 20411660

[mbt213980-bib-0248] Timmis, K. , and Hallsworth, J.E. (2022) The Darkest Microbiome: a post‐human biosphere. Microbial Biotechnol In press. 10.1111/1751-7915.13976. PMC8719803 34843168

[mbt213980-bib-0249] Tiwari, B.S. , and Tripathi, S.N. (1998) Water binding in sub‐aerial cyanobacteria. Indian J Biochem Biophys 35: 52–61. 9699420

[mbt213980-bib-0250] Tollefson, J. (2020) How hot will Earth get by 2100? Nature 580: 443–445. 32322083 10.1038/d41586-020-01125-x

[mbt213980-bib-0251] Tregoning, G.S. , Kempher, M.L. , Jung, D.O. , Samarkin, V.A. , Joye, S.B. , and Madigan, M.T. (2015) A halophilic bacterium inhabiting the warm, CaCl_2_‐rich brine of the perennially ice‐covered Lake Vanda, McMurdo Dry Valleys, Antarctica. Appl Environ Microbiol 81: 1988–1995. 25576606 10.1128/AEM.03968-14 PMC4345359

[mbt213980-bib-0252] Trembath‐Reichert, E. , Morono, Y. , Ijiri, A. , Hoshino, T. , Dawson, K.S. , Inagaki, F. , and Orphan, V.J. (2017) Methyl‐compound use and slow growth characterize microbial life in 2‐km‐deep subseafloor coal and shale beds. Proc Natl Acad Sci USA 44: E9206–E9215. 10.1073/pnas.1707525114 PMC5676895 29078310

[mbt213980-bib-0253] Tsutsaeva, A.A. , Anan’ina, A.E. , Balyberdina, L.M. , Stepaniuk, L.V. , and Pavlenko, N.V. (2008) Long‐term storage of industrial microbial strains. Microbiology 77: 621–624. 19004353

[mbt213980-bib-0254] Twizeyimana, M. , and Hartman, G.L. (2010) Culturing *Phakopsora pachyrhizi* on detached leaves and urediniospore survival at different temperatures and relative humidities. Plant Dis 94: 1453–1460. 30743399 10.1094/PDIS-02-10-0131

[mbt213980-bib-0255] Ulrich, N. , Nagler, K. , Laue, M. , Cockell, C.S. , Setlow, P. , and Moeller, R. (2018) Experimental studies addressing the longevity of *Bacillus subtilis* spores – The first data from a 500‐year experiment. PLoS One 13: e0208425. 30513104 10.1371/journal.pone.0208425 PMC6279046

[mbt213980-bib-0256] Vadell, M.V. , Salomone, V.N. , Castesana, P.S. , Morandeira, N.S. , Rubio, A. , and Cardo, M.V. (2021) Assessment of environmental hazards to public health in temperate urban Argentina. EcoHealth 18: 250–266. 34448975 10.1007/s10393-021-01535-x

[mbt213980-bib-0257] van der Wielen, P.W.J.J. , Bolhuis, H. , Borin, S. , Daffonchio, D. , Corselli, C. , Giuliano, L. , *et al*. (2005) The enigma of prokaryotic life in deep hypersaline anoxic basins. Science 307: 121–123. 15637281 10.1126/science.1103569

[mbt213980-bib-0258] de Vera, J.P. , Schulze‐Makuch, D. , Khan, A. , Lorek, A. , Koncz, A. , Möhlmann, D. , and Spohn, T. (2014) Adaptation of an Antarctic lichen to Martian niche conditions can occur within 34 days. Planet Space Sci 98: 182–190.

[mbt213980-bib-0259] Vertucci, C.W. (1989) The effects of low water contents on physiological activities of seeds. Physiol Plant 77: 172–176.

[mbt213980-bib-0260] Vertucci, C.W. (1990) Calorimetric studies of the state of water in seed tissues. Biophys J 58: 1463–1471. 19431782 10.1016/S0006-3495(90)82491-7 PMC1281098

[mbt213980-bib-0261] Vollmer, M. (2019) The freezing of lakes in winter. Eur J Phys 40: 035101.

[mbt213980-bib-0262] Voosen, P. (2021) Perseverance will explore history of ancient lake. Science 26: 870–871. 10.1126/science.371.6532.870 33632824

[mbt213980-bib-0263] Vreeland, R.H. , Rosenzweig, W.D. , and Powers, D.W. (2000) Isolation of a 250 million‐year‐old halotolerant bacterium from a primary salt crystal. Nature 407: 897–900. 11057666 10.1038/35038060

[mbt213980-bib-0264] Walker, V.K. , Palmer, G.R. , and Voordouw, G. (2006) Freeze‐thaw tolerance and clues to the winter survival of a soil community. Appl Environ Microbiol 72: 1784–1792. 16517623 10.1128/AEM.72.3.1784-1792.2006 PMC1393208

[mbt213980-bib-0265] Warren, S.G. , Brandt, R.E. , and Grenfell, T.C. (2006) Visible and near‐ultraviolet absorption spectrum of ice from transmission of solar radiation into snow. Appl Opt 45: 5320–5334. 16826269 10.1364/ao.45.005320

[mbt213980-bib-0266] Webb, S.J. (1965) Bound Water in Biological Integrity. Springfield, IL: Charles C. Thomas.

[mbt213980-bib-0267] Williams, T.J. , Liao, Y. , Ye, J. , Kuchel, R.P. , Poljak, A. , Raftery, M.J. , and Cavicchioli, R. (2017) Cold adaptation of the Antarctic haloarchaea *Halohasta litchfieldiae* and *Halorubrum lacusprofundi* . Environ Microbiol 19: 2210–2227. 28217912 10.1111/1462-2920.13705

[mbt213980-bib-0268] Winston, P.W. , and Bates, D.H. (1960) Saturated solutions for the control of humidity in biological research. Ecology 41: 232–237.

[mbt213980-bib-0269] Wright, R.J. , Clegg, R.J. , Coker, T.L.R. , and Kreft, J.U. (2020) Damage repair versus aging in an individual‐based model of biofilms. mSystems 5: e00018‐20. 33051374 10.1128/mSystems.00018-20 PMC7567578

[mbt213980-bib-0270] Yakimov, M.M. , Lo Cono, V. , La Spada, G. , Bortoluzzi, G. , Messina, E. , Smedile, F. , *et al*. (2015) Microbial community of seawater‐brine interface of the deep‐sea brine Lake *Kryos* as revealed by recovery of mRNA are active below the chaotropicity limit of life. Environ Microbiol 17: 364–382. 25622758 10.1111/1462-2920.12587

[mbt213980-bib-0271] Yashina, S. , Gubin, S. , Maksimovich, S. , Yashina, A. , Gakhova, E. , and Gilichinsky, D. (2012) Regeneration of whole fertile plants from 30,000‐y‐old fruit tissue buried in Siberian permafrost. Proc Natl Acad Sci USA 109: 4008–4013. 22355102 10.1073/pnas.1118386109 PMC3309767

[mbt213980-bib-0272] Yung, P.T. , Shafaat, H.S. , Connon, S.A. , and Ponce, A. (2007) Quantification of viable endospores from a Greenland ice core. FEMS Microbiol Ecol 59: 300–306. 17313579 10.1111/j.1574-6941.2006.00218.x

[mbt213980-bib-0273] Zaikova, E. , Benison, K.C. , Mormile, M.R. , and Johnson, S.S. (2018) Microbial communities and their predicted metabolic functions in a desiccating acid salt lake. Extremophiles 22: 367–379. 29350297 10.1007/s00792-018-1000-4

[mbt213980-bib-0274] Zolensky, M.E. , Bodnar, R.J. , Yurimoto, H. , Itoh, S. , Fries, M. , Steele, A. , *et al*. (2017) The search for and analysis of direct samples of early Solar System aqueous fluids. Philos Trans A Math Phys Eng Sci A 375: 20150386. 10.1098/rsta.2015.0386 PMC5394253 28416725

