# Supplementary material for: Water is a preservative of microbes
Source: Microb Biotechnol. 2021 Dec 22;15(1):191–214. doi: 10.1111/1751-7915.13980 (PMC8719826; doi:10.1111/1751-7915.13980)
Supplement: Supplementary file 1 — Appendix S1. Dating of fluid inclusions of halite. [file MBT2-15-191-s002.doc]

**Supporting Information**

**Water is a preservative of microbes**

John E. Hallsworth

*Institute for Global Food Security, School of Biological Sciences, Queen’s University Belfast, 19 Chlorine Gardens, Belfast, BT9 5DL, UK.*

For correspondence. E-mail: j.hallsworth@qub.ac.uk

**Contents:**

Dating of fluid inclusions of halite

**Dating of fluid inclusions of halite**

Primary fluid inclusions in chemical sediments such as bedded halite are known to form as the mineral first precipitates from surface waters, including lakes and seawater. Therefore, any microorganisms trapped in halite primary fluid inclusions are the same age as the host depositional halite. However, many previous studies of microorganisms in halite have not evaluated the geologic context (by using petrographic observations) to know whether the microbial cells were sampled from primary fluid inclusions or by secondary or pseudosecondary inclusions formed by later waters migrating through the halite. This has sometimes led to doubts about the age of those microbial cells (Hazen and Roedder, 2001; Powers *et al*., 2001; Pedrós-Alió, 2021). Although it can be difficult to determine the absolute age of a halite sample, it is relatively straightforward to determine whether the fluid inclusions are depositional (primary) or formed later (secondary). Primary fluid inclusions in halite formed as the mineral was precipitating from lake- or ocean water can be recognized via petrographic observations indicating that their age is the same as the host halite. The criteria for the identification of primary fluid inclusions in bedded halite formed at Earth’s surface as a chemical precipitate from a surface water as opposed to halite that forms diagenetically from groundwater) include:

- existence in a chevron, cornet, or cumulate crystal;

- location as part of a growth band in one of these crystal types; and

- cubic or sub-cubic crystal shape (Figure 1; Lowenstein and Hardie, 1985; Goldstein and Reynolds, 1994). Studies carried out by the research groups of K.C. Benison, T.K. Lowenstein, M.R. Mormile, and B.A. Schubert have involved syringing of individual fluid inclusions that are known to be primary (avoiding bulk crushing of halite that can mix primary and secondary fluid inclusions and potentially some soluble minerals trapped as solid inclusions). Whereas detrital siliciclastic grains in (young) bedded halite have been dated (Ku *et al*., 1998; Lowenstein *et al*., 2003), there are no reports of absolute dating of fluids in primary inclusions of bedded halite. More dating has not been done in part because samples are so small in volume, relatively few researchers have historically worked on halite, and absolute dating of ancient halite has not been considered a priority (because relative dating indicates geologic time periods).

Returning to the work of Schreder-Gomes and Benison (2021) and Schreder-Gomes *et al*. (2021), the authors are confident that the cells in the Precambrian fluid inclusions are themselves around 830 million-years-old because the fluid inclusions that host them meet the criteria for being depositional. Whereas secondary inclusions can migrate through the crystal structure of the salt (driven by temperature gradients) and leave the crystal structure unchanged (Shao *et al*., 2019; Hu *et al*., 2020), the primary inclusions of the 830 million-years-old halite had not (Schreder-Gomes *et al*., 2021). In general, ancient organisms can be dated using radiocarbon analyses. However, there are two difficulties that prevent carbon dating being performed on cells within fluid inclusions. First, carbon dating is only accurate for young material (less than ~70,000 years-old), and second, there is generally not enough organic material within fluid inclusions for carbon dating.

**References**

Goldstein, R.H., and Reynolds, T.J. (1994). *Systematics of Fluid Inclusions in Diagenetic Minerals.* SEPM (Society for Sedimentary Geology) Short Course 31.

Hazen, R.M., and Roedder, E. (2001) Biogeology. How old are bacteria from the Permian age? *Nature* **411:**155–156.

Hu, M., and Rutqvist, J. (2020) Finite volume modeling of coupled thermo-hydro-mechanical processes with application to brine migration in salt. *Comput Geosci* **24:**1751–1765.

Ku, T., Luo, S., Lowenstein, T., Li, J., and Spencer, R. (1998). U-Series chronology of lacustrine deposits in Death Valley, California. *Quat Res* **50:** 261–275.

Lowenstein, T.K., and Hardie, L.A. (1985), Criteria for the recognition of salt-pan evaporites. *Sedimentology* **32:** 627–644.

Lowenstein, T.K., Hein, M.C., Bobst, A.L., Jordan, T.E., Ku, T.L., and Luo, S. (2003) An assessment of stratigraphic completeness in climate-sensitive closed-basin lake sediments: Salar de Atacama, Chile. J Sediment Res **73:** 91–104.

Pedrós-Alió, C. (2021) Time travel in microorganisms. *Syst Appl Microbiol* **44:** 126227.

Powers, D., Vreeland, R., and Rosenzweig, W. (2001) How old are bacteria from the Permian age? *Nature* **411:**155–156.

Schreder-Gomes, S., Benison, K.C., and Bernau, J. (2021) 830-million-year-old microorganisms in primary fluid inclusions in halite. *Geology* In press.

Shao, H., Wang, Y., Kolditz, O., Nagel, T., and Brüning, T. (2019) Approaches to multi-scale analyses of mechanically and thermally-driven migration of fluid inclusions in salt rocks. *Phys Chem Earth* **113:** 1–13.
