# Supplementary material for: Water is a preservative of microbes
Source: Microb Biotechnol. 2021 Dec 22;15(1):191–214. doi: 10.1111/1751-7915.13980 (PMC8719826; doi:10.1111/1751-7915.13980)
Supplement: Supplementary file 2 — Appendix S2. Supporting Information (Szostak, 2003) [file MBT2-15-191-s001.doc]

**Supporting Information**

**Water is a preservative of microbes**

John E. Hallsworth

*Institute for Global Food Security, School of Biological Sciences, Queen’s University Belfast, 19 Chlorine Gardens, Belfast, BT9 5DL, UK.*

For correspondence. E-mail: j.hallsworth@qub.ac.uk

**Contents:**

Szostak, J.W. (2003) Explaining the universe without a clue. *Fitness of the Cosmos for Life Workshop October* *2003*. Cambridge, MA, USA: Harvard University.

**Explaining the Universe Without a Clue**

**by**

**Jack W. Szostak**

The statement that small changes to certain fundamental physical contents would drastically alter the structure of the Universe, rendering it unfit for Life as we know it, is so dramatic and striking that it demands closer scrutiny. In what sense is it valid to play with the values of the fundamental physical constants? When are these changes small enough that we should be surprised? These points have been cogently addressed by Manson (2000), who pointed out that the concept of fine-tuned physical parameters only makes sense if the actual values of those parameters are arbitrary, i.e. not constrained by physical law, and thus our observed values are essentially the result of a frozen historical accident. If that is so, and this is by no means certain, then we can ask what is the range of allowed values of a physical constant, and moreover what is the expected probability distribution that those values would take in independent origins of the Universe. Only knowing these facts can we even ask whether the physical constants that describe our particular Universe are likely or unlikely to fall in a range consistent with Life in the Universe. In other words, only then would we have any basis for being surprised at the actual values of the physical constants in our Universe. This probabilistic interpretation of fine-tuning seems very sensible, but provides severe challenges to the development of physical theory.

A probabilistic interpretation of fine-tuning also provides a useful approach to asking whether we should be surprised by any aspects of chemistry, biochemistry, or biological evolution that constrain the emergence of Life as we know it. The fundamental question then becomes, was the emergence of life on earth a singular event of extremely low probability, or a virtually inevitable consequence of the initial physical conditions (or something in between)? As in physics, this is a somewhat tenuous area of inquiry, because the extent of our ignorance is so great and the domain of our knowledge is so small. Nevertheless, I hope to show in this essay that there is at least the possibility that experimental research can address some of these issues and may ultimately lead to a clearer picture of whether the origin of life was a process of high or low probability. Coupled with advances in astronomy and astrophysics, this will ultimately allow us to make a more reasoned assessment of whether we are likely to find life elsewhere in our galaxy.

First, a brief word on what is meant by ‘Life as we know it’ is in order. Although there is no clear dividing line between life as we know it, and theoretically possible variations on life, most biochemists would agree that life as we know it means carbon based life in an aqueous environment. Thus, cell structure, metabolism and inheritance are based on an organic chemistry compatible with water. At finer levels of resolution, i.e. the specific bases that make up the genetic material, or the specific amino acids that are used to make proteins, it is easy to imagine alternatives that do not affect the fundamental properties of the resulting cells. However, despite much speculation, no detailed or convincing proposal for non-carbon and/or non-water based life has been made. Because we are only familiar with one biochemical kind of life, this may reflect more a limitation of our imaginations, rather than any real chemical or physical limitation. Indeed, I will propose experiments below to explore the feasibility of non-aqueous life.

**Water**

The remarkable properties of water have often been pointed out (Henderson, 1913) and are central to claims that chemistry is predisposed to the emergence of life. The unusual chemical and physical properties of water, together with the fact that all life that we know is aqueous life, lead to the common supposition that life may be impossible without water. Some authors have gone beyond this to speculate that life would be impossible given even small changes in the properties of water. The implication is presumably that the observed properties of water are so unlikely to have occurred ‘by chance’ that they must be the result of design or selection. But is this really the right way to look at the issue of why water appears so special to us? Surely, whatever the nature of the solvent that happened to lead to the emergence of life, that life would continuously adapt to that solvent as a result of strong and continuing selective pressures. After a short time, the solvent would appear to be pre-adapted to that form of life. This would be (and is in our case) simply an illusion resulting from the action of natural selection forcing life to adapt as well as possible to its environment. Of course, the chemistry of any non-aqueous life would be more or less different from our own, depending on how different the hypothetical solvent was from the water we know and love.

As a way of illustrating this point, let us look again at the properties of water, and ask ourselves, perhaps in a less biased way (or at least with a different bias), if water is really so ideal for life. Stepping back conceptually from our parochial water-dominated viewpoint, we can immediately see that water is really a noxious, toxic, corrosive and generally lethal environment for life. In fact given the well known properties of water one might almost be tempted to say that it’s a miracle that life ever began in such a solvent! Consider the chemical reactivity of water (and especially the nucleophilicity of its hydroxide ion). Hydrolytic reactions destroy biomolecules: as just one example, the spontaneous deamination of cytosine to uracil illustrates the fact that water is probably the most genotoxic substance known. Very elaborate and energetically expensive repair systems have evolved to compensate for the damaging effects of water on the genetic material.

Beyond this critical problem, huge numbers of metabolic intermediates are unstable in water. It is a commonplace observation that many metabolic enzymes bind their substrates in pockets designed to exclude water. Mutations that allow access of water to the active site are often found to lead to the synthesis of unwanted side-products. More generally, why do organic chemists do almost all of their elaborate syntheses in non-aqueous solvents? Because they use highly reactive starting materials, or reactions that involve unstable intermediates that would be instantly destroyed by contact with water. Therefore, water restricts the domain of chemistry that is suitable for life to a very small fraction of the possibilities that might otherwise be open to exploitation by evolution. The deleterious effects of water on pre-biotic chemistry are manifold, such as for example the short half-lives of sugars in water. The difficulty of polymerization of monomers to make nucleic acids and peptides is a consequence of the energetic cost of performing a dehydration reaction in water. Again and again we see that water is in fact the main culprit in making it difficult to see how life could have emerged on the early earth.

Our new view of water does not stop with chemical hydrolysis. The remarkable solvent power of water (due to its polarity, its resulting ability to solvate ionic species, and its ability to act as both an excellent hydrogen bond donor and acceptor) leads to a requirement for specialized cell membranes to act as compartment boundaries, so that the molecules of life, both metabolic and genetic, will not simply disperse. But this in turn has a host of sequelae, including the need for a chemical specialization to keep metabolic precursors soluble and yet unable to diffuse across the cell membrane. This problem is solved by the use of phosphate esters as tags for metabolic intermediates and as a key component of the genetic materials, as pointed out in the wonderful classic paper by Westheimer (1987) on why nature chose phosphates.

Since phosphate appears to be so special in allowing life to exist in water, we should look at it more carefully. In a ‘fine-tuned’ environment, one might expect that the one molecule that can salvage life from the ravages of water would be abundant and readily available in a suitable form. Instead, phosphate is far from abundant, and phosphorus is in fact the least abundant of the six major elements of life. Moreover, chemically reduced phosphorus is highly soluble in iron, and the vast majority of phosphorus disappeared long ago into the earth’s core during planetary differentiation, leaving the mantle and crust highly depleted in this essential element. Furthermore, most of the remaining traces of phosphorus, once it equilibrated with the more oxidized mantle chemistry, have precipitated as highly insoluble salts, most notoriously apatite. Thus life is left to the mercy of the traces of phosphate that have escaped these dominant chemical processes.

Yet another devastating aspect of the physical chemistry of water is the narrow temperature range in which liquid water is stable. This in fact defines the well known habitable zone - the narrow band surrounding a star in which liquid water would be stable on the surface of a planet. Once water freezes, its growing ice crystals destroy delicate cellular structures and rip membranes into shreds. It is only through advanced biochemistry resulting from continued selective pressure that Darwinian evolution has allowed certain organisms to evolve defenses against ice-crystal formation. Some organisms synthesize complex sugars such as trehalose to prevent the crystallization of water, such that low temperatures lead to a less damaging glassy state. Other organisms synthesize peptides that bind to the surface of ice crystals and prevent their continued growth. The adaptations of life to the dangerous properties of water are truly wonderful and varied.

Finally, consider that the amazing hydrogen bonding properties of water, so often cited, render the three-dimensional folding of proteins and nucleic acids almost impossible. The reason for this is that in the unfolded state, i.e. when an RNA or protein chain is extended and open to the solvent, all of its hydrogen bonding functional groups are satisfied by hydrogen bonding to water. There is little if any energy gained through the formation of intra-molecular hydrogen bonds during the folding process, since any two protein groups that are going to interact with each other must first be desolvated. One might conjecture that if water were not such a good H-bond donor and acceptor, it might be possible to generate stable folded structures from much shorter polymeric sequences, or perhaps from polymers built of simpler, easier to make building blocks. Indeed this point of view makes apolar solvents look at least in some ways much more hospitable to life than water. This is of course a conjecture that is subject to experimental test, through efforts to design new life forms that grow, replicate, evolve and adapt in non-aqueous solvents.

In light of the above considerations, it seems naïve to consider the properties of water as evidence for fine-tuning in the structure of our world. Rather, water has a long list of properties, some of which are favorable, and others of which are unfavorable both with respect to the origin of life and its continued existence. It is the fact that life has so successfully adapted, through the lengthy process of Darwinian evolution, to its aqueous environment, that makes water seem so favorable to life. Of course, Henderson was aware of this, and carefully phrased his claims by saying ‘life as we know it’, implying that other forms of life might be possible in environment with limited or no free water. However, this possibility, although frequently raised in the context of wild speculation about life elsewhere in the universe, remains almost unexplored in any serious way.

The design of non-aqueous living systems is a fascinating possibility. Part of the problem of course is that those few of us working in this area are still preoccupied with the design of more conventional water-based life forms. This project, while still a large task involving many engineering-design issues, does not seem to face any major conceptual barriers. There is a spirit of confidence, resulting from recent progress, that self-replicating genetic polymers can indeed be evolved in the laboratory, that self-replicating compartment structures can be designed, and that compatible conditions can be found that will result in supra-molecular systems that exhibit all the qualities of cellular life including the ability to evolve and adapt to their environment. Given this state of the art, it seems appropriate to speculate briefly on the possibility of some day extending this experimental paradigm to the design of non-aqueous life forms. There seem to be no fundamental barriers to the design of genetic systems that would functional in apolar environments. Indeed the work of Rebek has already demonstrated how the principles of shape and hydrogen bonding complementarity can be extended to simple models of templated synthesis in non-aqueous solvents (Tjivikua 1990). The possibility of forming cell-like compartments in such solvents is less explored, but even here we have a base of understanding in the design of reverse micelles - tiny compartments with a polar interior shielded from the apolar exterior. It does not seem too great a leap to imagine the design of appropriate amphiphilic molecules that would make inside-out bilayer membranes (with polar interiors and apolar exteriors) that could form membrane bound vesicles in apolar solvents. Once we begin to think about specific issues, the experimental challenges and opportunities associated with the design of living systems in non-standard environments seem to beckon like the proverbial (but water-based) sirens.

**Sequence Space and Fitness Landscapes**

Having established that such a fundamental aspect of our chemical environment of water is biased neither for nor against the appearance of life, let us turn to the thornier question of our more abstract environment, sequence space. Sequence space is an abstract multi-dimensional space whose coordinate axes simply list the component monomers that make up a polymer such as RNA or DNA or protein. It may be considered to be simply the collection of all possible sequences. There is a common misconception that the vastness of sequence space implies some difficulty in finding useful sequences, and especially in finding those special sequences that would allow life to begin. The number of possible RNA or protein sequences is indeed enormous, and a collection of all possible protein sequences of length 100 amino acids would have far more mass than that of the visible universe. And yet, here we are, with genomes coding for thousands of proteins, many far larger than that. How did we get here, in the sense of ‘here’ being our coordinates in sequence space?

The simple answer is that the immensity of sequence space is irrelevant to this question, and that what is important is simply the fraction of all possible sequences that are capable of carrying out some useful function. These functional sequences may be easy or hard to find (or to evolve) depending on how common they are. Unfortunately this is not generally something that we know much about. In fact it is very difficult to put any reasonable quantitative constraints on this question. Our ignorance is vast, and the experimental results are few. However, there are a few observations that taken together might lead us to think that the problem of finding functional sequences may not be so dire. First of all, the amount of genomic sequence data available to us is growing rapidly. This database allows one to compare gene and protein sequences across essentially all of phylogeny. Even a cursory glance at the sequence alignment for any given protein will show that enormous sequence variation is tolerated - thus even for a given enzymatic activity catalyzed by a protein with a specific folded shape, there are a very large number of sequences that will generate that structure and that function simply because most positions in a protein don’t matter that much, or else changes at one place are easily compensated for by changes somewhere else. When one considers that a given function, such as the catalysis of a particular chemical reaction, could be carried out by many distinct proteins with distinct shapes and chemical mechanisms, then the number of possible sequences that are consistent with a given function becomes even greater. For example, we know that there are at least five distinct independent families of proteases. What we don’t know is how many such distinct protein families might be possible, but are not (or have not yet) been found in biology. This number could also be enormous, in which case functional sequences might be so common as to not present much of a barrier to the emergence of function from random search processes, such as mutation coupled with natural selection.

This is an issue that we and others have been attempting to address experimentally by generating large collections of random sequence RNA or protein molecules, and then looking to see if we can find functional molecules in those collections. This work began over ten years ago with the development of methods for selecting functional molecules from large pools of random RNA molecules. We were able to show that RNA sequences that would fold up into specific three-dimensional shapes with binding sites for small molecules were easily accessible in such experiments. Thus, RNAs that specifically recognize many small molecule targets seem to be present at abundances of roughly 1 in 10^10-11^ (i.e. one in ten to a hundred billion), and since we can make 10^15^ such random molecules in even a small scale lab experiment, the interesting RNAs that can recognize target molecules are rare but still easily detectable. Similar experiments suggest that many catalytic RNAs are similarly abundant (but see below for caveats on replicating RNAs). Can these kinds of experiments be extended to proteins? Several recent technological developments do in fact allow one to search for functional proteins starting from essentially random sequence protein libraries. However, these experiments are a lot harder to do. So far, my laboratory has done one such experiment and the result is surprisingly similar to the earlier RNA work - finding functional proteins doesn’t seem to be drastically easier or harder than finding functional RNAs. We were able to isolate a small set of distinct, independent proteins, all of which bind ATP. As far as we can tell, they have no sequence similarity to any known biological protein. What is the meaning of this experiment? My interpretation is that functional proteins are common in sequence space, to the point that if we can do it in the lab, it would be easy for nature to find new functional proteins by evolution (for example, by searching sequence space through random drift followed by selection of useful sequences). On the other hand, I was amused to be told that our work had shown up within hours of publication in the creationist web literature, cited as providing experimental proof that proteins could never have emerged by natural evolutionary processes because they are too rare!

Even if we agree that solutions to many simple biochemical functions are easy to find in sequence space, the situation is more difficult when we consider the origin of life. Let us accept for the sake of argument that the beginnings of cellular life required some sort of self-replicating RNA molecule, i.e. an RNA replicase, an RNA molecule that is a good enough RNA polymerase to replicate itself. The probability that such a sequence could form by chance within a reasonable amount of random RNA is an extremely important question: if any possible self-replicating RNA is too rare to be found by chance, then either the origin of life was itself an event of very low probability, or life began in some other way. Unfortunately, we do not yet have any self-replicating RNA, so we cannot answer this question directly. The most interesting molecule to be evolved in the laboratory so far is an RNA molecule with limited RNA polymerase ability (it can copy up to 14 nucleotides of a template RNA strand - see Johnston et al. 2001). However, this ribozyme is fairly large at almost 200 nucleotides in length, so significant further improvement in RNA polymerase activity is required before we can hope to see it copy molecules of its own sequence. Such molecules are likely to incorporate additional domains of structured RNA, and therefore to be even larger. The complexity of this RNA is such that an origin by chance in one step is unlikely in the extreme. However, before jumping to the conclusion that life could not have started in this way, several other factors should be considered. First of all, there may be other RNAs that are simpler and better, and these may be found in subsequent experiments. Second, the conditions used to evolve the original ribozyme and then to optimize it may themselves have been highly sub-optimal. The addition of other factors such as different metal ions or short peptides or other cofactors might make it much easier to find shorter simpler RNA replicases. Thirdly, this ribozyme uses nucleoside triphosphates as substrates - these are the substrates used to make nucleic acids in modern biology, and may not have been relevant at earlier stages in the origin of life. To address this, we are currently attempting to evolve RNA polymerases that use more chemically reactive substrates.

The above arguments apply to an RNA based origin of life, but it is by no means certain that RNA was the first genetic polymer. It is certainly possibly that some progenitor nucleic acid existed (perhaps Eschenmoser’s threose based TNA, see Schoning, 2000), that was easier to form chemically and easier to replicate. Again, experiments are possible to begin to test these ideas. We are currently using laboratory evolution methods to try to evolve the molecular tools that we need to replicate TNA sequences, so that we can then evolve TNA sequences that carry out biochemical functions such as binding and catalysis. In principle such experiments may allow the comparison of the functional capabilities of many different polymers, and may give us a new appreciation for whether or not our particular polymers are special.

TNA is only one potential progenitor of RNA in the origin of life. Alternatives have been proposed, and others are no doubt out there waiting to be discovered. And no doubt chemists will argue passionately for years to come that this one is pre-biotically plausible while that one is not. In the end, the experiments will be done, and we will have a better understanding, based on facts, and we may (or may not) be able to propose a plausible pathway leading to the origin of life. Perhaps we will have a super-abundance of plausible pathways, so that any actual pathway seems contingent of historical accident. Or perhaps we will be left with only one, and that one path so fraught with difficulty that our presence seems a miracle. But at present, our areas of ignorance are so vast that it is impossible to say whether the origin of life on earth was a virtual certainty or a unique fluke. Given this, it would seem to be more productive to avoid holding passionately to either view, and instead direct our energies to trying to solving this puzzle.

**Deterministic vs. Stochastic Outcomes of Evolutionary Processes**

Given the immensity of sequence space, both for nucleic acids and proteins, one might think that the chance of similar sequences being selected independently to carry out the same function would be vanishingly small. However, this intuitive reaction is incorrect, for the same reasons discussed above with respect to finding useful sequences in the first place. In the simplest case, we can imagine some useful molecular task that can be carried out by a very short, simple sequence (or more generally, an abundant family of related sequences). This sequence may for example specify a particular structure that we recognize as an enzyme (in the case of proteins) or ribozyme (in the case of RNA). Now, if we make the further assumption that all other ways of carrying out this molecular function involve structures that are significantly more complex, then purely by chance the simplest and most likely sequence (and its corresponding structure) will evolve again and again, independently. Exactly this kind of phenomenon has been seen in several examples of laboratory experiments in molecular evolution. For example, repeated selection in different laboratories have converged on the same simple RNA motif that folds into a three-dimensional shape that recognizes and binds to ATP. Similarly, if one selects for RNA sequences that cleave themselves at a biologically useful rate, one will observe the repeated independent selection of sequences that fold into the so-called hammerhead motif (Salehi-Ashtiani, 2002). In this case we know that there are other motifs that can self-cleave, but it appears that the hammerhead motif is the simplest and therefore most common such motif in RNA sequence space. The implications for biological evolution are clear. We see the hammerhead motif in many very distantly related organisms (and their viral parasites). The possibility that these organisms have evolved the hammerhead motif independently must be taken seriously, although this conclusion will be difficult to prove.

Having shown that convergent molecular evolution can take place, we can begin to ask how common this phenomenon might be. Is it indeed so common that we can conceive of biological evolution as a largely deterministic search for the simplest solutions to a defined series of biochemical problems, themselves universal due to the universality of chemistry? Here matters become more complex. A consideration of the protein structures used in biology has led some to conclude that the protein folds are a limited set of possible structures determined by chemical laws (Denton, 2002) - and that all evolution does is to search out and find these Platonic forms. The reason that this is a viable hypothesis is that each of the common protein folds can be specified by an enormous number of distinct sequences - in other words, finding these forms by random search processes is simply not that difficult a task. What is the number of distinct protein folds? Structural analysis of biological proteins reveals that many proteins that carry out different functions use the same basic folded structure (for example, the α−β barrel fold is used by hundreds of distinct enzymes). This structural analysis may be converging on an estimate of a few thousand distinct folds. It is important to realize that more folds continue to be discovered almost daily, so the end result is uncertain. However, there is a larger issue that cannot be addressed by the analysis of biological proteins alone, and that is whether biology is revealing to us a small subset of all possible protein folds, or alternatively, whether we are seeing that biology uses essentially all of the possible folds. Biology might very well use just a subset of the possible folds either as a result of historical accidents (i.e. contingent on the sequences that were sampled early in evolution), or because a subset of the folds have some advantages not shared by other folds. In my opinion this remains an open question, subject to experimental investigation.

Returning for the moment to laboratory explorations of RNA sequence space, it is instructive to contrast the results of selections for RNA motifs that bind the similar biomolecules ATP and GTP. As mentioned above, a single simple structural motif (encoded by a family of related sequences) dominates selections for ATP binding. The same kind of experiment done with GTP as the target however leads to the isolation of numerous distinct RNA structures. There is no one dominant winner. Thus the ATP-directed selection appears deterministic, in that repeated independent selections converge on one solution, whereas GTP selections appear to have stochastic outcomes, depending on the random, contingent variables such as the initial sampling of sequences.

There is another intriguing possibility that has been raised as a result of the analysis of some RNA selection experiments. In some cases, the structures that emerge to satisfy the imposed selective pressure are quite complex, and calculations of the probability that such a structure would be present in the initial library of random sequences lead to absurdly low values such as one in a billion. This was just the case in an RNA selection experiment carried out in my laboratory some ten years ago (Bartel 1993) - we obtained a new, interesting ribozyme, but subsequent study of the ribozyme led to an apparent paradox. We should never have obtained such a complex molecule in the first place! Of course, we could have just been lucky - but subsequent similar experimental findings suggest that something deeper is at work. We think that there may be enormous numbers of distinct complex ribozymes all capable of carrying out the selected function. Any one of these structures is in fact too complex to have much chance of appearing in any particular experiment, but in aggregate, finding some solution is quite likely. That solution will appear to be rare, but that rarity simply reflects the other unseen structures, which together make the evolution of some structure altogether unremarkable. This hypothesis, which I should emphasize is still unproven, makes the clear experimental prediction that repeated selections carried out under the same conditions should always yield different complex solutions.

A similar situation is likely to apply with proteins, but here the experimental tests are even more difficult and further from realization. Nevertheless, it seems reasonable that the number of distinct protein folds may increase rapidly with complexity. The number of complex folds may be truly vast, implying that biology can and does sample only a small fraction of these possibilities. In approaching this problem, we must be careful to specify in advance what we mean by a distinct fold, for there is a danger that the argument could otherwise degenerate into semantic squabbling. The reason is that simple folds may be seen as substructures of more complex folds - in the limit, all folds are built from simpler substructures, and where to draw the line is not always clear.

To summarize this argument, biology has a strong tendency to modify an existing structure rather than invent a new one - simply because a successful modification is more likely to arise by chance than a new, complete and highly effective structure. This is likely to be as true at the molecular level as at the morphological level. Since the initial molecular structures are likely to be small and simple, the number of ‘choices’ available may be small, favoring a reproducible or deterministic aspect to the biological evolution of new structures. However, the above discussion is based on an implied constancy of conditions and molecular structures. We know that small changes in molecular structure, such as for example the presence or absence of the single hydroxyl group that defines RNA as different from DNA, can have large effects on the outcome of laboratory evolution experiments, where small changes often lead to completely different macromolecular structures that are in turn defined by completely different sequences. Thus if the environmental or chemical conditions on some other planet were such as to favor a slightly different chemistry for genetic molecules, or for proteins, perhaps a different basis set of nucleotides or amino acids or a small change in backbone chemistry, then we would expect that virtually all molecular structures would in fact be different. Thus the molecular beginnings of life could be driven in different directions by being very sensitive to the initial conditions. Whether convergence at higher levels of structure and organization would subsequently occur remains a tantalizing possibility, but one that will be difficult to test by either experiment or exploration in the near future.

Finding unexpectedly rare RNA structures in laboratory evolution experiments may be a metaphor for apparent fine-tuning on larger scales - our observed solution appears so unlikely that, perfectly reasonably, we search for an explanation. The answer could be simply good fortune (perhaps explained by the anthropic principle), or, more likely, it could be that there are simply so many different solutions that overall, finding one is more likely than not. What would this mean with respect to the presence of life in the Universe? At this time it appears, at least to some scientists, that the essential qualities of our Universe that render it suitable for the presence of life are defined by a set of physical parameters that are unconstrained by theory, and hence are arbitrary. Furthermore, it is supposed that the choice of our particular felicitous set of parameter values is so unlikely as to beg explanation. But recall (and see Manson) that we lack a higher order physical theory that could tell us anything about how likely or unlikely these parameter values actually are. Even if our Universe is based on a parameter set of low probability - how can we know what fraction of alternative possibilities would be compatible with Life? We are far from being able to calculate the detailed properties of our world from first principles (otherwise we would not need so many experimental physicists!), and we are that much further from being able to calculate the implications of randomly chosen physical constants especially when they are all changed at once, and not simply varied one at a time by small increments. By (distant) analogy with searches of RNA and protein sequence space, might not the parameter space defining alternative Universes be populated with a great many distinct combinations compatible with Life?

**Living in a Probabilistic Universe**

Perhaps the most philosophically and emotionally difficult transition in the scientific worldview has been the transition from the deterministic outlook of the Newtonian ‘Clockwork Universe’ to the probabilistic thinking in which modern science is so deeply embedded. This transition began not with quantum mechanics but with the earlier development of the statistical treatment of thermodynamics, a development that was fiercely resisted at the time, not least by its primer mover, Boltzmann. Nowadays we see our Universe as pervaded by probabilities. The fundamentals of quantum mechanics can tell us the likelihood that an atomic nucleus will decay in the next minute or the chance that an electron will scatter in this direction or that, but can never tell us what will happen to a specific particle at a particular time. And yet, when enough of these uncertainties are averaged over a long enough period of time, we see our orderly, predictable macroscale surroundings, explained in the limit of large numbers by classical physics. There is a certain irony in the fact that our most mechanistic and deterministic beliefs are founded on a seething bedrock of randomness and chaos. This underlying foundation of probabilities is always there, and its implications reach throughout all of science. In biology, the relatedness of all earthly life is evident in the patterns of nucleotides that compose every genome. And yet the sources of the variation that lead genomes to change from generation to generation are fundamentally random, an unending sequence of undoubted historical accidents. But as in physics, so in biology, order emerges from chance and even at the molecular level, genetic sequences can converge on the most likely solutions again and again. Its time to move beyond the debate between the dominance of determinism or contingency and recognize that both forces play a role and have to be incorporated into any comprehensive view of evolutionary history.

**Bibliography**

Bartel, D.P., Szostak, J.W. Isolation of new ribozymes from a large pool of random sequences. Science (1993) 261:1411-1418.

Davis, J.H. and Szostak, J.W., Isolation of high-affinity GTP aptamers from partially structured RNA libraries. Proc. Natl. Acad. Sci. USA (2002) 99:11616-11621.

Denton, M.J., Marshall, C.J. and Legge, M. The protein folds as Platonic forms: New support for the pre-Darwinian Conception of Evolution by Natural Law). J. Theor. Biol. (2002) 219: 325-342.

Henderson, L.J. The Fitness of the Environment. 1913. Macmillan Co.

Johnston, W.K., Unrau, P.J., Lawrence, M.S., Glasner, M.E. and Bartel, D.P. RNA-catalyzed RNA polymerization: Accurate and general RNA templated primer-extension. Science (2001) 292:1319-1325.

Manson, N.A. Inquiry (2000) 43:341-52.

Salehi-Ashtiani, K. and Szostak, J.W. In vitro evolution suggests multiple origins for the hammerhead ribozyme. Nature (2001) 414:82-84.

Schoning, K. –U.; Scholz, P.; Guntha, S.; Wu, X.; Krishnamurthy, R.; Eschenmoser, A. Chemical etiology of nucleic acid structure: The α-threofuranosyl (2’-3’) oligonucleotide system. *Science* **2000**, *290*, 1347-1351.

Tjivikua, T. ,Ballester, P. and Rebek, J. , Jr. A Self-replicating system. J. Am. Chem. Soc. (1990) 112: 1249-1250.

Westheimer, F. H. Why Nature Chose Phosphates. Science (1987) 235:1173.
